# A cytosolic carbonic anhydrase molecular switch occurs in the gills of metamorphic sea lamprey

**DOI:** 10.1038/srep33954

**Published:** 2016-10-05

**Authors:** D. Ferreira-Martins, S. D. McCormick, A. Campos, M. Lopes-Marques, H. Osório, J. Coimbra, L. F. C. Castro, J. M. Wilson

**Affiliations:** 1 Centro Interdisciplinar de Investigação Marinha e Ambiental (CIIMAR/CIMAR) Universidade do Porto, 4050-123, Porto, Portugal; 2 Instituto de Ciências Biomédicas Abel Salazar, Universidade do Porto, 4050-313, Porto, Portugal; 3 USGS, Leetown Science Center, S.O. Conte Anadromous Fish Research Laboratory, 01376, Turner Falls MA USA; 4 i3s-Instituto de Investigação e Inovação em Saúde, Universidade do Porto, 4200-135, Porto Portugal; 5 Instituto de Patologia e Imunologia Molecular da Universidade do Porto, 4200-135, Porto, Portugal; 6 Departamento de Patologia e Oncologia, Faculdade de Medicina, Universidade do Porto, 4200-319, Porto, Portugal; 7 Departamento de Biologia, Faculdade de Ciências, Universidade do Porto, 4169–007, Porto, Portugal; 8 Department of Biology, Wilfrid Laurier University, N2L 3C5, Waterloo, Canada

## Abstract

Carbonic anhydrase plays a key role in CO_2_ transport, acid-base and ion regulation and metabolic processes in vertebrates. While several carbonic anhydrase isoforms have been identified in numerous vertebrate species, basal lineages such as the cyclostomes have remained largely unexamined. Here we investigate the repertoire of cytoplasmic carbonic anhydrases in the sea lamprey (*Petromyzon marinus*), that has a complex life history marked by a dramatic metamorphosis from a benthic filter-feeding ammocoete larvae into a parasitic juvenile which migrates from freshwater to seawater. We have identified a novel carbonic anhydrase gene (*ca19*) beyond the single carbonic anhydrase gene (*ca18*) that was known previously. Phylogenetic analysis and synteny studies suggest that both carbonic anhydrase genes form one or two independent gene lineages and are most likely duplicates retained uniquely in cyclostomes. Quantitative PCR of *ca19* and *ca18* and protein expression in gill across metamorphosis show that the *ca19* levels are highest in ammocoetes and decrease during metamorphosis while *ca18* shows the opposite pattern with the highest levels in post-metamorphic juveniles. We propose that a unique molecular switch occurs during lamprey metamorphosis resulting in distinct gill carbonic anhydrases reflecting the contrasting life modes and habitats of these life-history stages.

The sea lamprey, *Petromyzon marinus*, Linnaeus 1758 is a basal vertebrate characterized by a complex anadromous life cycle. The larvae or ammocoetes are benthic, freshwater filter feeders that undergo a dramatic morphological and physiological transformation into parasitic feeders that migrate downstream to the sea. At the end of the marine trophic phase adults re-enter fresh water and migrate upstream to spawn and then die^1^,^2^.

Carbonic anhydrase is a zinc metalloenzyme, primarily involved in the reversible hydration/dehydration reactions with CO_2_, thus involved in CO_2_ transport and ionic and acid–base regulation^3^. Although carbonic anhydrases are found in all animals, in vertebrates only the α-carbonic anhydrase family is present^4^,^5^,^6^. In mammals, carbonic anhydrases are categorized according to their subcellular localization. Carbonic anhydrase isoforms 1, 2, 3, 7 and 13 form the functional cytosolic group^7^ and CA5 orthologues are mitochondrial^8^. The remaining carbonic anhydrase isoforms are membrane-associated with an extracellular orientation (4, 9, 12, 14, 15), secreted (6) or non-catalytic (8, 10, 11) (reviewed by Gilmour and Perry^9^).

The teleost cytosolic carbonic anhydrase gene repertoire is notably different from that of mammals^9^. Phylogenetic analyses indicate that teleosts retained an ancestral state of a single high activity carbonic anhydrase isoform, in contrast with the carbonic anhydrase gene expansion and functional segmentation in mammals^9^ and we have renamed this clade as *ca17*. In teleost fishes the *ca17b* (blood type; *ca2-like b* or *ca2b*) has been found to be mainly expressed in red blood cells (RBC) in zebrafish^10^ and trout^11^,^12^. In contrast, *ca17a* (*cac* or *ca2-like a*) has higher expression in gill than kidney and RBC^10^,^12^. More recently, cytoplasmic carbonic anhydrases have been cloned and characterized from gills of the Antarctic fishes *Trematomus eulepidotus*, *Trematomus lepidorhinus, Trematomus bernacchii* and *Cygnodraco mawsoni*^13^. Cytosolic carbonic anhydrases have also been cloned from blood samples of various non-teleost fishes such as the holostean gar, (*Lepisosteus osseus)*^14^, the elasmobranch dogfish, (*Squalus acanthias)*^15^, and from cyclostomes such as lamprey, *P. marinus*^16^, and the Pacific hagfish, *Eptatretus stouti*^17^. To date only a single carbonic anhydrase isoform has been characterized in sea lamprey gill tissues and blood^16^.

For acid-base regulation, fishes rely on metabolic compensation, which involves the exchange of acid-base equivalents directly from their external environment: H^+^ and HCO_3_^−^ are exchanged for Na^+^ and Cl^−^, respectively. Cytosolic carbonic anhydrase is an essential component of this process, providing an intracellular pool of H^+^ and HCO_3_^−^ from CO_2_ hydration for these exchange processes in ion and acid-base regulatory epithelia such as the gill and kidney^9^. Although RBC carbonic anhydrase is central to transport of CO_2_ as HCO_3_^−^ in the plasma in most vertebrates, the lack of functionally significant Cl^−^/HCO_3_^−^ exchange (band 3 protein) in lamprey RBCs limits transport to intracellular RBC HCO_3_^− ^18.

In the present study, we tested the hypothesis that metamorphosis, which marks a dramatic change in the life style and physiology of sea lamprey, requires changes in cytosolic carbonic anhydrase expression. A similar shift has already been documented in amphibian development and has been postulated to be related to distinct life stage strategies and physiological challenges (see review from Tufts *et al*.^19^). To address the hypothesis we cloned and sequenced a novel ammocoete carbonic anhydrase orthologue (*ca19*) and together with the previously described carbonic anhydrase (*ca18*)^16^ elucidated changes during metamorphosis. Expression levels of the carbonic anhydrase lamprey genes were determined at the transcript level using quantitative RT-PCR, and protein level using immunoblotting and further characterized by MALDI-TOF mass spectrometry and molecular modeling. Adaptive changes following salinity exposure that would occur following the normal downstream migration of sea lamprey were also explored.

## Results

### Diversity and evolution of cytosolic CA isoforms in vertebrates

We began by examining the repertoire of carbonic anhydrase sequences in the sea lamprey genome (www.ensembl.org, Pmarinus_7.0). Following that, through PCR we were able to identify a previously unreported carbonic anhydrase gene in this species. The novel carbonic anhydrase sequence was identified in the ammocoete gill. The new transcript is 1786 bp with an ORF of 771 bp and 5′ and 3′ UTR of 87 and 928 bp, respectively. The transcript codes for a 257 amino acid (aa) protein that has 67.5% aa identity with the published carbonic anhydrase isoform from lamprey and 55.9% to 57.4% and 51.3% to 57.8% with rainbow trout and human Cac (Ca17a) and CA2 isoforms, respectively (Supplemental Fig. 1). In order to examine possible functional differences in the isoforms, we analyzed the amino acid residues of the active site of the novel lamprey isoform where CO_2_ binding and proton shuttling occur (Table 1). We showed that the newly sequenced gene was most similar to the existing sea lamprey carbonic anhydrase isoform Ca18 and *O. mykiss* Ca17a with respectively 7 and 8 of 36 aa residue substitutions. This analysis also showed a change at the aa level of a substrate associated pocket at position 207 with a valine (very hydrophobic) substituted for an arginine (hydrophilic) (Table 1). In addition, *in silico* analysis performed on carbonic anhydrase cytosolic isoforms showed Ca18 and Ca19 had electrostatic potentials of −8.0 and −2.0, respectively (Supplemental Table 6).

**Figure 1:**
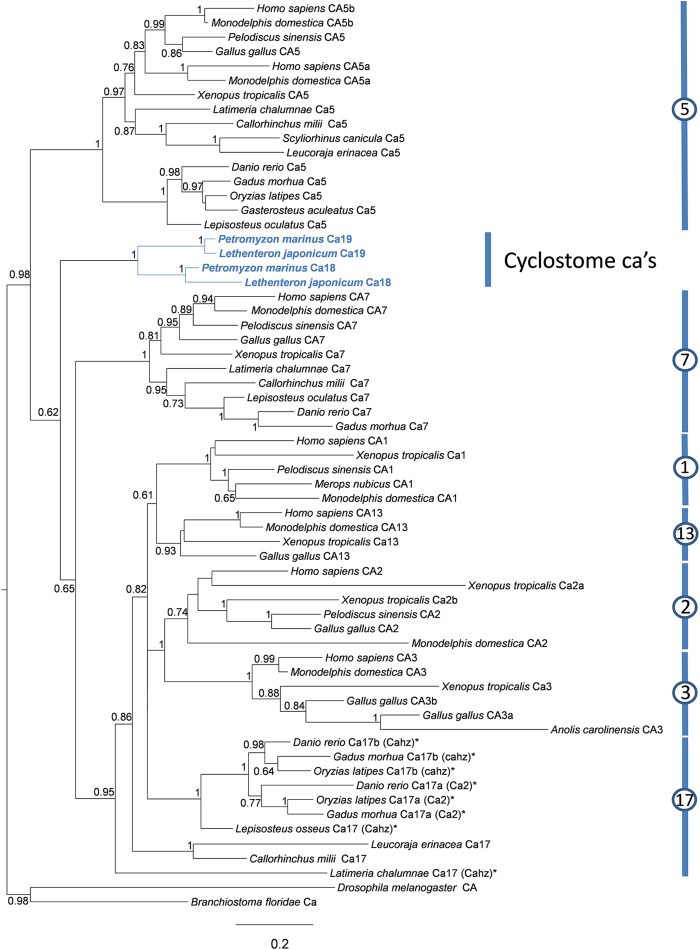
Maximum likelihood phylogenetic tree describing relationships among carbonic anhydrase proteins from representative vertebrate *taxa*. Node values represent branch support using a Bayes algorithm (values below 0.5 are not shown). Asterisk indicates zfin.org or ensemble.org name. Accession numbers for all sequences are provided in the supplementary material.

We next undertook phylogenetic analyses to clarify the orthology of the collected lamprey carbonic anhydrase sequences (Fig. 1). We also re-examined the evolutionary relationships of intracellular carbonic anhydrase genes in vertebrate species. Our analysis identifies four well-supported clades: *CA5*, *CA7*, a third assembly composed of tetrapod *CA1/2/3/13* and non-tetrapod *ca17* genes, and a fourth clade with the two lamprey sequences. In support of our findings in the sea lamprey, we also detected the presence of these two sequences in the recently released genome sequence of the Japanese lamprey^20^ (Fig. 1). *CA5* genes are found in all of major vertebrate lineages including chondrichthyans, teleosts and tetrapods. Although a *CA5*-like sequence was found in the lamprey genome it was not included in the present analysis (see materials and methods). *CA7* genes are also found in the basal gnathostome lineage the chondrichthyans. The novel lamprey carbonic anhydrase gene strongly groups with a previously described carbonic anhydrase sequence to form a distinct clade basal to gnathostome *CA7* and *CA1/2/3/13/17* (Fig. 1). Based on this analysis we rename the sea lamprey carbonic anhydrase described by Esbaugh and Tufts^16^ *ca18* and the novel sequence carbonic anhydrase *ca19*. The present analysis also provides some insight into the duplication timing and origin of *CA1/2/3/13/17* isoforms. Despite the poor statistical support in some internal nodes within the *CA1/2/3/13/17* clade, the combination with genome mapping information of these genes in various gnathostome lineages, allows the proposal that the expansion of the *CA1/2/3/13* clade took place after the divergence of coelacanth from tetrapods (Fig. 1; Supplemental Fig. 2). Thus, *ca17* “one-to-one” orthologues are found in chondricthyans, coelacanth and gar with two teleost-specific genome duplicates, *ca17a* and *ca17b*. Moreover, we find a further carbonic anhydrase gene unique to amphibians, named *ca2b*, and an additional *CA3* in birds, named *CA3b*.

The finding that the two lamprey cytosolic isoforms group together and are at the base of the gnathostome *CA1/2/3/13/17* and *CA7* clades complicates a conclusion over their true orthology. To provide further insights into their origin, we examined the genomic locations of the lamprey carbonic anhydrase genes and compared them with those of their human counterparts (Supplemental Fig. 3). However, this information is available for the lamprey *ca5* and novel cytosolic carbonic anhydrase but not the previously described cytosolic carbonic anhydrase^16^. The human cytosolic carbonic anhydrase genes localize to genomic regions related by genome duplication, the so-called 2R, at linkage group 3^21^. In the case of the lamprey, we find that the ortholog of the gene close to *ca5* (*heatr3*) maps to human chromosome 16 (Supplemental Fig. 3), providing strong support of its orthology. In contrast, the human orthologues of the genes in the vicinity of the lamprey novel cytosolic carbonic anhydrase do not localize to either chromosome 8 or 16 as would be expected if this gene was a true ortholog of either *CA1/2/3/13* or *CA7*, respectively. However, we find some clues that indicate that the lamprey novel cytosolic carbonic anhydrase might represent a retained paralogue resulting from 2R, but subsequently lost in gnathostomes. In effect, two genes, *c14orf119* and a novel *ccne* gene indicate that this genomic region in lamprey is probably orthologous of a region of the human genome in chromosome 14 which is paralogous to the regions in chromosome 8 and 16 (Supplemental Fig. 3). Considering these observations and the phylogenetic results, we suggest that the lamprey cytosolic carbonic anhydrases might represent novel carbonic anhydrase gene lineages resulting from genome duplications in vertebrate ancestry, which have been retained uniquely in the cyclostomes lineage. We thus propose calling the previously described^16^ and novel cytosolic carbonic anhydrases *ca18* and *ca19*, respectively, in line with the naming of new carbonic anhydrases. The recent proposal of a single genome duplication in the vertebrate ancestor would imply a different interpretation of our data^22^. Thus, additional mapping and phylogenetic data from other cyclostome species should help to clarify these issues.

### Tissue distribution

**Figure 2:**
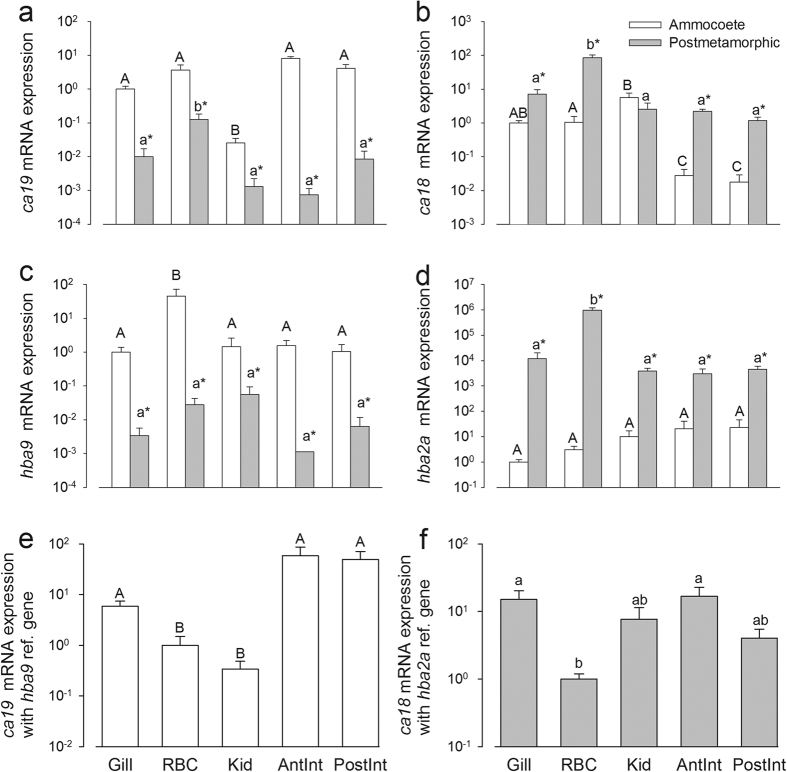
Relative mRNA expression (mean ± S.E.M.) of the novel carbonic anhydrase isoform (**a**) *ca19*, (**b**) *ca18* (GenBank AAZ83742), (**c**) putative larval hemoglobin *(hba9)*, (**d**) postmetamorphic hemoglobin (*hba2a*) in *P. marinus* ammocoete and post-metamorphic juvenile gill, blood, kidney, anterior and posterior intestine determined by qPCR. In the final two panels (**e**) *ca19* and (**f**) *ca18* are expressed using *hba9* and *hba2a* as respective reference genes and rescaled relative to RBC groups. Tissue expression levels are significantly different if they lack common letters [ammocoetes (uppercase) and juvenile (lowercase)]. Significant differences between ammocoete and juvenile within a given tissue are indicated by an asterisk. Two-way ANOVA and SNK post-hoc test P < 0.05 (N = 4).

At the mRNA level, the cytosolic carbonic anhydrase isoform *ca19* was expressed in ammocoete gill, blood, and gut (anterior and posterior intestine) at similar levels with significantly lower expression in the kidney (Fig. 2a). In post-metamorphic juveniles, RBC had relative higher mRNA expression than the other tissues tested but *ca19* levels were otherwise significantly lower than in any ammocoete tissue tested. The mRNA expression of *ca18* was significantly higher in all post-metamorphic lamprey tissues except kidney (Fig. 2b). Blood had the highest mRNA expression levels of *ca18* in post-metamorphic lamprey with no significant differences between the other tissues. In ammocoetes, *ca18* mRNA expression was significantly higher in kidney, with intermediate expression in gill and blood and the lowest expression in gut (anterior and posterior intestine). RBC’s contamination in each type of tissue was assessed by the analysis of the corresponding mRNA expression of hemoglobin in tissue samples. Sea lamprey hemoglobin *hba2a (=aHb2a)* showed the highest mRNA expression levels in post-metamorphic juvenile RBC’s with similar lower levels (<1%) in the other tissues (Fig. 2d). In ammocoetes, mRNA expression levels of *hba2a* were significantly lower than in postmetamorphic juveniles. In contrast, in ammocoetes the putative larval hemoglobin *hba9 (=aHb9)* mRNA levels were significantly higher in blood (>50-fold) compared to all other tissues (Fig. 2c), indicating that blood was unlikely to contribute to observed differences in tissue carbonic anhydrase mRNA levels. The *hba9* mRNA levels were also significantly lower in post-metamorphic juveniles than in ammocoetes (Fig. 2c). In addition, *ca19* and *ca18* expression were calculated using *hba9* and *hba2a* as respective reference genes and tissue mRNA levels expressed relative to RBC groups to further assess RBC contamination (Fig. 2e,f, respectively). In ammocoetes, the gill and gut (anterior and posterior intestine) *ca19* was significantly higher than RBC (and kidney). In post-metamorphic lamprey, *ca18* levels were significantly higher in gill and anterior intestine only. The mRNA expression levels of the reference genes *gapdh* and *18s* were found not to be consistent across the tissues tested (Supplemental Fig. 4).

### Changes in cytosolic carbonic anhydrase mRNA levels during metamorphosis and increasing salinity acclimation of post-metamorphic juveniles

**Figure 3:**
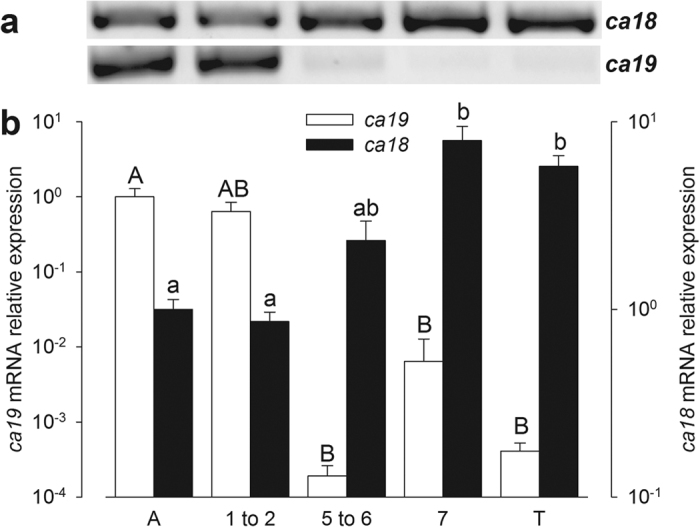
Relative mRNA expression (mean ± S.E.M.) of *P. marinus ca18* and *ca19* in ammocoete (A; N = 16), metamorphosis stages 1 to 2 (N = 12), 5 to 6 (N = 5), 7 (N = 3) and post-metamorphic (T; N = 8). Cropped representative bands from qPCR reactions from the same run are shown above. Changes in *ca18* and *ca19* are analyzed separately and bars with like letters are not significantly different from each other (in lower and upper case letters, respectively). Two-way ANOVA and SNK post-hoc test P < 0.05.

Higher branchial transcript levels of the novel *ca19* isoform were found in ammocoetes compared to post-metamorphic juveniles and adults. Relative mRNA expression levels of sea lamprey *ca18* and *ca19* isoforms in gill tissue were assessed using real time RT-PCR (Fig. 3a,b). The analyses indicated that the *ca19* isoform is almost exclusively expressed in the ammocoete and during the 1^st^ and 2^nd^ stages of metamorphosis. Low levels of expression of *ca18* were found during these stages, which increased significantly from stage 5 onward during metamorphosis. Post-metamorphic juveniles show an increase of *ca18* mRNA expression in response to increased salinity (Supplemental Fig. 5).

### Immunoblotting and 2-DE analysis

**Figure 4:**
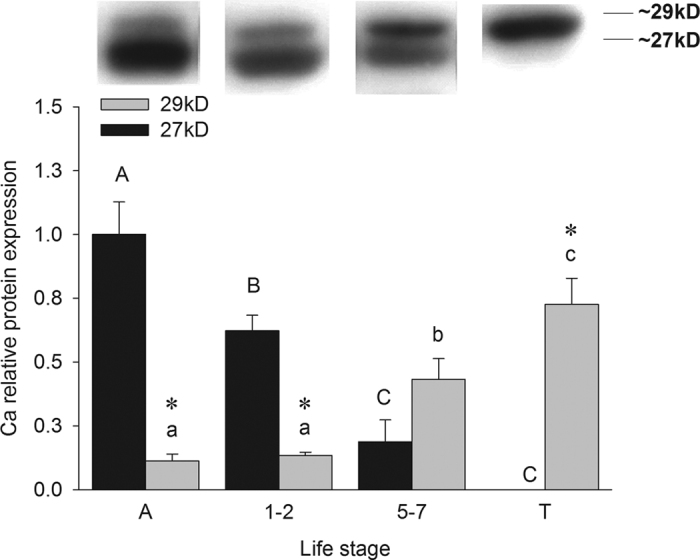
Representative cropped western blot of sea lamprey gill carbonic anhydrase expression (mean ± S.E.M.) using a heterologous cytosolic carbonic anhydrase antibody (1:2000) with crossreactive 27 kD and 29 kD bands in a developmental stage specific pattern collected under identical experimental conditions. The developmental series includes ammocoete (N = 10); metamorphic stages 1 to 2 (N = 20); stages 5 to 7 (N = 7); and post-metamorphic juvenile (N = 15). Changes in 27 kD and 29 kD bands are analyzed separately and bars with like letters are not significantly different from each other (in upper and lower case letters, respectively). Significant differences between 27 kD and 29 kD bands within a developmental group are indicated by an asterisk. See supplemental Fig. 8 for original blots. Two-way ANOVA and SNK post-hoc test P < 0.05.

Probing of lamprey gill immunoblots with a heterologous mammalian CA2 antibody revealed the presence of two immunoreactive bands at 27 and 29 kDa, respectively (Fig. 4). A significant difference in band expression was found in ammocoetes, metamorphic stages 1 to 2 and juveniles. Expression of the 27 kDa band was highest in ammocoetes and stages 1–2 and decreased significantly during metamorphosis and was undetectable in post-metamorphic juveniles. In contrast the 29 kDa protein band expression was significantly lower in ammocoetes and stages 1–2 and increased significantly at the latter stages of metamorphosis (stage 5–7, post-metamorphic juvenile). Consequently, protein expression of the 27 kDa band was significantly greater than the 29 kDa band in ammocoetes and early metamorphic stages while no significant differences were found in protein band expression for stages 5 to 7. In post-metamorphic juveniles protein expression of the 29 kDa band was significantly greater than the 27 kDa band. To confirm the identity of the two bands, a proteomics approach was taken. The 2-DE gel immunoblots probed with the heterologous cytosolic carbonic anhydrase antibody (Supplemental Fig. 7) indicated spots of potential interest. The 2-DE gel spots were examined by MS/MS and database search (Supplemental Fig. 6, Table 5). High protein identification scores revealed the presence of two Ca18 (Ca18-i, Ca18-ii) in RBC’s of ammocoetes and post-metamorphic lamprey (Supplemental Fig. 6A) and six Ca19 spots (Ca19-i, Ca19-ii, Ca19-iii, Ca19-iv, Ca19-v and Ca19-vi) in the RBC’s of ammocoetes only (Supplemental Fig. 6B). In agreement with the 1D western blots, the six Ca19 spots corresponded to the 27 kDa band and the Ca18 spots to the larger 29 kDa band. The theoretical pI values for Ca18 and Ca19 were estimated at 5.51 and 6.51, respectively, with a prediction of 20 and 19 probable modifications by phosphorylation each (ProMoST^23^. Supplemental Table 7), with predicted pI values ranging from 4.64–5.39 and 5.05–6.27. The NetPhos 3.1 Server analysis predicted 27 and 34 potential phosphorylation sites for Ca18 and Ca19, respectively (Supplemental Table 8; Supplemental Fig. 1). The respective non-phosphorylated pI values using Compute pI/Mw tool were 5.47 and 6.23.

## Discussion

We have identified and characterized a novel cytosolic carbonic anhydrase isoform, *ca19*, which is highly expressed in the gill and RBCs of sea lamprey during its early life history stages. In addition we have compared this novel isoform with the cytosolic carbonic anhydrase (*ca18*) previously described in adult sea lamprey by Esbaugh and Tufts^16^. Our findings indicate a clear and sustained isoform switch during metamorphosis. The *ca19* isoform is expressed during the ammocoete stage and during the initial stages of metamorphosis. In contrast, *ca18* mRNA and protein is expressed at low levels during the larval stage and becomes more highly expressed only during the latter stages of metamorphosis and into the post-metamorphic stages when the sea lamprey is prepared to enter its marine trophic phase^2^. These results suggest *ca19* isoform may play an important role during the time sea lamprey live in the silty substrates of freshwater streams when the ammocoete is hyperosmoregulating and lives as a filter feeder on a low energy diet.

The carbonic anhydrase gene family has a wide diversity of isoforms and functions among fish groups^9^. However, our understanding of carbonic anhydrase diversity, function and distribution is still incomplete when considered in an evolutionary framework. Prior to this study a single high-activity cytosolic carbonic anhydrase isoform (*ca18*) had been found in the sea lamprey, although the evolutionary relationships to other vertebrate carbonic anhydrases is contentious^9^,^15^,^16^. This isoform was found in a variety of tissues including blood, brain, kidney and gill but absent in muscle, liver and intestine in adult lamprey using Northern blotting^16^. In our study we have identified a novel cytosolic carbonic anhydrase, isoform 19, which is preferentially expressed in the lamprey’s larval stages in tissues such as gill, blood and anterior and posterior intestine. The discrepancy in intestinal expression between the two studies can be explained by the differences in the life history stages and the sensitivity of the techniques that were used. Esbaugh and Tufts^16^ studied adults on their upstream spawning migration, and the digestive system is known to degenerate as lampreys do not feed at this stage and die after spawning^24^. Northern blotting is also less sensitive than PCR based techniques used in the present study^25^,^26^. In contrast, our findings of *ca18* and *ca19* mRNA expression indicate the presence of cytoplasmic carbonic anhydrases in the gut during larval and post metamorphic stages, when the digestive system is fully functional. In addition, the hematopoietic tissue in larval lamprey is in the gut associated typhosol^27^,^28^ and thus high carbonic anhydrase mRNA expression would be expected^29^. As for aHb expression, which we used as an indicator of RBC contamination of tissues, our results indicate a switch from *aHb9* to *aHb2a* in the ontogeny of sea lamprey in agreement with a recent report^30^.

The molecular mass of Ca19 determined *in silico* closely matched the values measured experimentally by gel electrophoresis. This supports our findings indicating changes in protein levels through immunoblotting from gill tissue probed with a heterologous CA2 antibody. The 27 kD band shows higher relative protein levels during earlier life-history stages, while during metamorphosis there is a shift in the protein expression of both bands. In the final metamorphic stages the 29 kD band is much more intense while the 27 kD band decreases and is undetected in fully transformed lampreys. Notably, the predicted molecular masses of Ca19 and Ca18 correspond to the 27 and 29 kDa bands, respectively. However, in order to confirm the reactivity of both isoforms with the heterologous CA2 antibody we utilized an MS/MS analysis approach of the excised 2-DE gel spots. This approach confirmed a match with the previously published carbonic anhydrase sequence (Ca18) by Esbaugh and Tufts^16^ in the RBC’s of post-metamorphic lamprey and the carbonic anhydrase sequence identified with this work (Ca19) in RBC’s of ammocoetes. The pI of eukaryotic proteins provides insight into their subcellular localization^31^ and experimental pI can be applied to distinguish protein isoforms and modifications^32^.

In most cases the pI predicted by the databases closely matches the experimentally determined value^33^,^34^,^35^, nevertheless it is not uncommon to experimentally observe shifts in these value. These shifts are due to protein modifications such as truncations and deletions but are more often associated with co - or posttranslational phosphorylation. Phosphorylation plays a key role in regulatory mechanisms in cells^36^,^37^,^38^ by replacing neutral hydroxyl groups on serine, threonine or tyrosine residues with phosphate group(s) that are negatively charged^39^. As a result, phosphorylation typically induces an acidic shift in pI. In agreement, analysis using ProMoST demonstrated that all experimental pI’s were found lower than their respective nonphosphorylated theoretical pI value. In addition to phosphorylation, the multiple Ca18 and Ca19 protein spots in this study may result from pI shifts attributed to acetylation at the N-terminal of the residues sequence. This has already been documented for acidic and neutral protein^33^, where the removal of amino group(s) by this process results in an adjustment of the acid-base balance and shifts the protein pI value.

Previous phylogenetic analyses have shown that fish cytoplasmic carbonic anhydrase diverged prior to the gene duplication events that gave rise to the mammalian carbonic anhydrase gene cluster^11^,^12^,^14^ with the exception of CA7, where orthologues have also been found in fish^16^. In mammals both high and low turnover carbonic anhydrase isozymes can be found while only high turnover isozymes are present in fish and are catalytically akin to mammalian CA2^40^. However, we were able to identify a new cytoplasmic carbonic anhydrase in lamprey. Our findings suggest that cyclostomes retain a unique cytoplasmic carbonic anhydrase set, since both phylogenetics and synteny analysis indicate that *ca18* and *ca19* might not be orthologues of previously described carbonic anhydrase genes in gnathostomes. The examination of the carbonic anhydrase genomic *loci* genes in lamprey and humans supports the hypothesis that duplications in vertebrate ancestry were instrumental in the elaboration of carbonic anhydrase diversity. We hypothesize that the cyclostomes’ cytoplasmic carbonic anhydrase genes probably represent retained genome duplicate paralogues. In effect, the lamprey carbonic anhydrase genes might represent genome duplicates of a different origin to gnathostomes. It has recently been put forward that vertebrates underwent one genome duplication, in contrast to the two or three rounds previously proposed^20^,^41^, which does not allow us to determine the exact duplication event from which cyclostome *ca18* and *ca19* arose.

An analysis of the Ca19 active site pocket revealed seven and eight amino acid differences from Ca18^16^, and rainbow trout Ca17a(cac)^11^,^12^,^19^,^42^,^43^, respectively, both of which have high catalytic rates. In comparisons with the mammalian low turnover CA1 and high turnover CA2, the Ca19 isoform shows twelve and nine amino acid differences from their respective active site pockets. However, for the most part the amino acid differences are predicted to be substitution neutral, and significantly the essential proton shuttle histidine-64 (H) and zinc binding ligand residues (H94, H96, H119) are conserved. However, two of the amino acids that border or have side chains projecting into the active site at positions 91 and 204 show predicted unfavourable substitution ratings (−2 and −3, respectively). At residue 91 conformationally flexible glycine (G) is found in place of hydrophobic isoleucine (I), and at residue 204 polar asparagine (N) is found in place of hydrophobic leucine (L). These and the other neutrally predicted amino acid differences might impart changes in the three dimensional structure^44^ and access to the catalytic site potentially impacting Ca19 activity, kinetics and/or inhibitor binding^45^. Analysis using NetPhos 3.1 Server demonstrated that five possible phosphorylation sites correspond to active site pockets of Ca19 (tyrosine-7 (Y), serine-29 (S), S67, threonine-200 (T) and T244 represented in Table 1), which are likely to alter the catalytic activity of the enzyme as demonstrated in rainbow trout by Carrie and Gilmour^46^.

In our analysis of the electrostatic potential of cytosolic carbonic anhydrases in fishes, we find that RBC carbonic anhydrases have values around 1.0 with the exception of zebrafish Ca17b (cahz), whereas tissue carbonic anhydrases have negative electrostatic potentials that tend to be more variable. Marino *et al*.^45^ observed that both *C. hamatus* and trout tissue type Ca17a shared a similar negative electrostatic potential in contrast to RBC Ca17b carbonic anhydrases suggesting a diversification of fish isoforms based more on cell type than species. These differences may reflect the dominate roles of RBC and tissue carbonic anhydrases in blood CO_2_ transport, and ion and acid-base regulation, respectively, related to protein interactions. Although both lamprey Ca18 and Ca19 have been demonstrated to be expressed in RBC, they both have negative electrostatic potential values which may reflect the different mode by which lamprey RBCs participate in the convective transport of blood gases^18^.

Cytosolic carbonic anhydrases in the gill are important for whole animal ion and acid-base regulation providing an intracellular pool of H^+^ and HCO_3_^−^ from CO_2_ hydration for exchange with Na^+^ and Cl^−^, respectively, as well as for aiding in metabolic processes and acid-base regulation of individual gill cells that have a high metabolism and generate excessive CO_2_ levels^5^. Branchial cytosolic carbonic anhydrases apparently do not have a role in facilitating respiratory CO_2_ elimination^47^. We propose that loss of the novel ammocoete isoform *ca19* is associated with the loss of the enigmatic ammocoete mitochondrion-rich cells (MRC) and that the increase in *ca18* is triggered by metamorphosis as preparation for the higher activity marine trophic phase of this species’ lifecycle.

In ammocoetes, Conley and Mallatt^47^ have localized carbonic anhydrase activity by enzyme histochemistry to the lamellar epithelium and RBCs. Significantly, this lamellar epithelial localization of carbonic anhydrase corresponds to the location of the ammocoete MRCs which are unique to the ammocoete stage and are lost during metamorphosis^3^,^48^. This pattern of loss mirrors that of *ca19.* The ammocoete MRCs make up ~60% of epithelial cells covering the gill lamellae and are mitochondrion-rich. The function of ammocoete MRC’s is unknown although various hypotheses have been presented that include ion uptake and metabolic waste elimination. The high mitochondrial density of these cells would indicate a high metabolic activity and cytosolic carbonic anhydrase would have a role in modulating cellular acid-base demands^3^. A role in ion regulation seems unlikely since Bartels and co-workers^49^ found no morphometric changes with ion poor water challenges and these cells are not present in adult freshwater migrants which also need to hyperosmoregulate. In addition, the ammocoete gill has very low NKA activity and neither NKA nor V-ATPase co-localize to these cells^50^. Instead it is the intercalated mitochondria-rich cells (IMRC) that express these ATPase that are predicted to drive ion regulatory processes^50^,^51^.

In addition, we postulate that the environmental conditions ammocoetes live under may shape their carbonic anhydrase expression profile. Ammocoetes live buried in the silt and muddy substrate^1^,^2^ where higher CO_2_ levels and humic substances contribute to a more acidic environment^52^. In substrate dwelling sand eel, Behrens *et al*.^53^ have demonstrated that O_2_ levels in the surrounding substrate drop and given that CO_2_ is eliminated during the exhalation, it would be reasonable to expect that CO_2_ levels would be higher. In trout, hypercapnia has been shown to increase branchial carbonic anhydrase (activity, protein and mRNA)^54^,^55^,^56^. While it is purely speculative at this point, the presence of *ca19* during the ammocoete life stages might impart a higher CO_2_ tolerance as an adaptation to this environment.

Branchial IMRC, which are expressed in other life history stages, are a more likely candidate for active ion uptake and acid-base regulation^3^,^54^ and we would propose as a site for the expression of *ca18*. Carbonic anhydrase immunoreactivity has been localized to IMRC using heterologous antibodies in freshwater post-metamorphic *Geotria australis*^51^ and sea lamprey^50^; however, in the latter study weaker immunoreactivity was also found throughout the rest of the gill epithelium. In both IHC studies, a similar apical localization with H^+^-ATPase was observed which supports the role of carbonic anhydrase as a provider of an intracellular supply of H^+^ for the pump through the carbonic anhydrase catalysed hydration of CO_2_^48^,^57^. In addition, results from the salinity acclimation of post metamorphic juveniles revealed an increase in *ca18* mRNA expression suggesting that this isoform plays an important role in adaptation to higher salinity environments as well. However, it should be noted that Reis-Santos *et al*.^50^ did not observe a change in the pattern of CA-immunoreactive cells with salinity acclimation. Nonetheless, increasing expression of *ca18* isoform at the mRNA and protein levels during metamorphosis may reflect the preparation for seawater entry and/or development of the parasitic stage in seawater. In a number of studies on teleost fishes (*Oreochromis mossambicus*^58^ *Fundulus heteroclitus*^59^, *Dicentrarchus labrax*^60^) gill carbonic anhydrase increases with salinity acclimation, although others have found that distribution of carbonic anhydrase does not change (reviewed by Conley and Mallatt^47^). The high metabolic activity of these ionocytes would require cytosolic carbonic anhydrase to alleviate any cellular acid-base disturbances from endogenously generated CO_2_. Such a role for carbonic anhydrase has been demonstrated in the shark salt secreting rectal gland by Shuttleworth and co-workers^61^ which perform the same function as seawater fish gill ionocytes.

## Conclusions

Our study provides insight into the molecular events that occur during the life history of sea lamprey. Specifically, we demonstrate a previously unknown molecular switch between carbonic anhydrase isoforms occurring during metamorphosis. We propose that the novel switch from the *ca19* to *ca18* is of functional importance and related to differences in the demands of ion and acid-base regulation and altered metabolic demands from adoption of an active blood-feeding lifestyle. However, clearly this is an area in need of further study, in particular the functional characterization of the catalytic, kinetic and inhibitor binding of Ca19.

## Materials and Methods

### Animals

*Petromyzon marinus* (L.) ammocoetes were collected by electrofishing from tributaries of the River Minho during the summer and autumn of 2011 and the Fort River MA USA during the summer of 2010. Animals collected from the Fort River MA, USA were sampled in the field (see Reis-Santos *et al*.^50^ for more details) and those from River Minho were transported to the Interdisciplinary Centre of Marine and Environmental Research, University of Porto, Portugal (CIIMAR, UP) and maintained in a large tank with dechlorinated tap water and mechanic and biologic filtration, and provided a sandy silt substrate. Water temperature was maintained at 15 °C. Animals were fed with a suspension of yeast twice a week and acclimated to these tank conditions for at least one week before experimentation. Animals were treated in accordance with the Portuguese Animal Welfare Law (Decreto-Lei no.197/96) and U.S. Geological Survey institutional guidelines, and animal protocols were approved by CIIMAR/UP and DGV (Ministry of Agriculture) and IACUC.

### Metamorphic Series and Salinity Experiment

For the metamorphosis experiment, 16 ammocoetes, nine stage 1, three stage 2, one stage 5, six stage 6, three stage 7 and eight post-metamorphic juveniles staged according to Youson and Potter^62^ were sampled from the Fort River MA, USA. Animals were sampled as described below.

Salinity acclimation of post-metamorphic juveniles was done as described in Reis-Santos and co-workers^50^ using Fort River lamprey.

### Sampling

Animals were killed with an overdose of ethyl-m-amino benzoate (MS-222 1:5000 buffered with sodium bicarbonate, pH 7.8 1:5000). Total length (mm), mass (±0.01 g) and stage were recorded for each animal. Blood samples were collected from the caudal vessels using a heparinized capillary tube after caudal transection, centrifuged and hematocrit recorded. Separated plasma and RBCs were snap frozen in liquid nitrogen and stored at −80 °C. Gill, kidney, anterior intestine, and posterior intestine were excised and snap frozen in liquid nitrogen and stored at −80 °C for further use.

### RNA isolation and PCR

Total RNA was isolated from tissue samples, quality assessed, quantified and converted to cDNA for PCR. Zebrafish carbonic anhydrase primers were used to isolate an initial partial sequence that was completed by RACE. Real-time PCR was conducted using *ca18* and *ca19* specific primers. [See Supplemental Materials and Methods for more details].

### Phylogenetic Analysis

Carbonic anhydrase sequences were collected from various genome databases such as Ensembl, GenBank or JGI (Joint Genome Institute) through Blastp searches. We also searched species-specific genome sites (e.g. http://esharkgenome.imcb.a-star.edu.sg/) to complete the screening of carbonic anhydrase gene diversity in vertebrates. Our analysis included all major vertebrate lineages with a total of 63 sequences (Accession numbers and Ensembl codes shown in the Supplemental Table 4). An ortholog of *CA5* from lamprey was not included in the analysis because it caused long-branch attraction in the tree (not shown). Amino acid sequences were aligned using the MAFFT software 7 with default parameters^63^. The alignment was stripped of all columns containing gaps leaving 152 positions for phylogenetic analysis. A Maximum Likelihood tree was constructed at PhyML (http://www.atgc-montpellier.fr/phyml/) protein evolutionary model was calculated in PhyML using smart model selection resulting in LG + G + I. Branch support was estimated using the aBayes method^64^ as implemented in PhyML. Trees were visualized with FigTree (v1.4.2; http://tree.bio.ed.ac.uk/software/figtree/).

### Immunoblotting

Gill tissue from the metamorphic series from ammocoete to juvenile stages were analyzed by immunoblotting as described in Reis-Santos and co-workers^50^ with modifications. Ten μg of sample were loaded onto polyacrylamide gels (10% T solving gels; 4% T stacking) and transferred to nitrocellulose membranes (Amersham (TM) Hybond (TM) ECL, GE Healthcare). Following blocking with 5% blotto, membranes were probed with a heterologous rabbit anti-bovine cytosolic CA polyclonal antibody (1:2000, Abcam Cambridge UK)^65^ or mouse anti-β-actin monoclonal (1:500; Sigma-Aldrich) overnight at room temperature. Membranes were then rinsed with TTBS (0.05% Tween-20 in Tris Buffered Saline, pH 7.4) and incubated for 1 hour with a goat anti-rabbit or anti-mouse IgG secondary antibodies conjugated to horseradish peroxidase, diluted in TTBS (1:50,000). Signal was obtained by enhanced chemiluminescence (ECL) with Millipore Immobilon Western chemiluminescent HRP substrate (Millipore Corporation, MA USA). Images were acquired using a luminescent image analyzer Fujifilm LAS-4000 mini and image reader software LAS-4000 version 2.0. Intensity of band signal was quantified using image analysis software Multi Gauge v3.1 (Fujifilm Tokyo, Japan). After detection, membranes were stripped with low pH stripping buffer and reprobed with other antibodies.

### Two-dimensional electrophoresis (2DE), MALDI-TOF/TOF analysis, protein identification and modeling

Since both cytosolic carbonic anhydrase isoforms are expressed in RBCs and due to the ease of blood collection we performed proteomic analysis using RBCs. RBC samples (150 μL) from post-metamorphic juveniles and ammocoetes were prepared for two-dimensional electrophoresis followed by MALDI TOF/TOF analysis and protein identification as described in Campos and co-workers^66^ and homology modeling [See Supplemental Materials and Methods for more details]. Predicted phosphorylated forms of Ca18 and Ca19 were determined *in silico* using NetPhos 3.1 Server (http://www.cbs.dtu.dk/services/NetPhos/; 67), ProMoST (http://prometheus.brc.mcw.edu/promost/; 23) and Compute pI/Mw tool according to Gasteiger *et al*.^68^ (ExPASy Server; http://web.expasy.org/compute_pi/).

### Statistical analysis

Data are presented as means + standard error of the mean. Statistical differences in mRNA and protein expression between tissues and life stage groups were determined using two-way ANOVA followed by the *post-hoc* Student-Newman-Keuls (SNK) test. One way ANOVA and SNK tests were performed on ammocoete and juvenile *ca19* and *ca18* normalized with their respective hemoglobin genes and juveniles exposed to different salinities. The statistic program SigmaPlot 11.0 was used for all analyses (Systat Software, Inc., GmbH, Germany). The fiducial limit was set at P < 0.05.

## Additional Information

**How to cite this article**: Ferreira-Martins, D. *et al*. A cytosolic carbonic anhydrase molecular switch occurs in the gills of metamorphic sea lamprey. *Sci. Rep.* **6**, 33954; doi: 10.1038/srep33954 (2016).

## Supplementary Material

Supplementary Information

## Figures and Tables

**Table 1:** Comparative analysis of carbonic anhydrase putative active site pocket amino acid residues and amino acids whose side chains either project into or border the active site in lamprey (Pma) Ca18 (GenBank;.AAZ83742.1) and Ca19 (GenBank, ALM25804.1), with rainbow trout (Omy) Cac (Ca17a) (GenBank, NP_001166020.1), and human (Hsa) CA1 (GenBank, NP_001729.1) and 2 (GenBank, NP_000058.1) modelled after Tashian *et al*. 4 and Gilmour *et al*. 15.

	7	29	61	62	64	65	66	67	69	91	92	94	96	106	107	117	119	121	131	141	143	145	192	194	198	199	200	201	202	204	206	207	209	211	244	246
	*	*			*			*			*	*	*	*	*	*	*							*		*	*						*		*	*
					+							Z	Z				Z	~		~	~				~							~	~			
*Hsa* CA2	Y	S	N	N	H	S	F	N	E	I	Q	H	H	E	H	E	H	V	F	L	V	G	W	Y	L	T	T	P	P	L	C	V	W	V	N	R
*Pma* Ca19	.	.	.	S	.	.	.	S	D	G	.	.	.	.	.	.	.	.	.	.	.	.	.	.	.	.	.	.	.	N	S	A	.	.	T	P
*Pma* Ca18	.	.	.	.	.	.	.	S	.	K	.	.	.	.	.	.	.	.	.	.	.	.	.	.	.	.	.	.	.	F	S	.	.	.	.	.
*Omy* Ca17a	.	.	.	.	.	.	.	Q	T	K	.	.	.	.	.	.	.	.	.	.	.	.	.	.	.	.	.	.	.	.	S	.	.	.	.	.
*Hsa* CA1	.	.	.	V	.	.	.	H	N	F	.	.	.	.	.	.	.	A	L	.	.	.	.	.	.	.	H	.	.	Y	S	.	.	I	.	.

The aa were aligned using ClustalW (BioEdit 7.0.9.0). Numbers represent the location of each amino acid relative to alignment with human CA2. *Active site aa residues; Z, zinc binding ligand; + , proton shuttling associated ligand; ~, substrate associated pocket.
