# Supplementary material for: A cytosolic carbonic anhydrase molecular switch occurs in the gills of metamorphic sea lamprey
Source: Sci Rep. 2016 Oct 5;6:33954. doi: 10.1038/srep33954 (PMC5050428; doi:10.1038/srep33954)
Supplement: Supplementary Information [file srep33954-s1.doc]

**A cytosolic carbonic anhydrase molecular switch occurs in the gills of metamorphic sea lamprey. D. Ferreira-Martins, S.D. McCormick, A. Campos, M. Lopes-Marques, H. Osório, J. Coimbra, L.F.C. Castro, J. M. Wilson**

**Supplemental Materials & Methods**

**RNA isolation, quantification and cDNA synthesis**

Total RNA was extracted using Aurum^(TM)^ Total RNA Mini Kits according to the manufacture’s recommendations (Bio-Rad, Hercules, CA, USA). Homogenization was performed using a bead mill Precellys 24 (Bertin Technologies, Montigny-le-Bretoneux, France) at 6400 rpm for 2 cycles of 15 s with a 5 s interval. Total RNA concentration and purity was determined spectrophotometrically (Nanodrop Thermo Fisher Scientific, USA), integrity assessed by agarose gel electrophoresis and cDNA synthesized from 1μg of total RNA with iScript cDNA Synthesis Kit (Bio-Rad) using a Doppio thermocycler (WVR International Ltd). The cDNA samples were stored at -20°C.

**RT-PCR, Real-time RT-PCR and sequencing**

PCR reactions were performed using cDNA from gill tissue using either DyNAzyme II DNA Polymerase (Thermo Scientific) or GoTaq® DNA polymerase (Promega, Madison, USA) according the manufacturer’s directions. Primers were designed using Primer3 ^69^ and were initially tested for specificity by RT-PCR. Initial PCR reactions were performed using a set of primers designed from zebrafish (*Danio rerio*) carbonic anhydrase mRNA sequence (*zfCA_F1* and *zfCA_R1*). All primers are described in Supplemental Table 1 and cycling profiles in Supplemental Table 2.

PCR products were separated on 2% agarose TBE (Tris-borate-EDTA) gels to confirm size of amplicons. All gels were stained with GelRed and images acquired with a Fujifilm LAS-4000 Mini luminescent image analyzer (Fujifilm Tokyo, Japan). A single band of the correct predicted size (327bp) was cut and cleaned using Illustra GFX PCR DNA and Gel Band Purification Kit (GFX column, GE Healthcare, Carnaxide, Portugal) and directly sequenced (StabVida, Oeiras, Portugal).

The product was confirmed as a cytosolic carbonic anhydrase by tBLASTx. From the resulting sequence two sets of primers were designed for nested RACE reactions; 3'RACE: forward (*PmCA_F1* and *PmCA_F2*) and 5'RACE reverse primer (*PmCA_R1* and *PmCA_R2*). RACE reactions were performed using SMARTer™ cDNA Amplification Kit (Clontech, California, USA) and Phusion Flash (Thermo Fisher Scientific) master mix. Cycling profiles are provided in Supplemental Table 2. The resulting PCR products were isolated and ligated into pGEM-T easy vectors (pGEM®-T Easy Vector Systems, Promega) and positive colonies were grown in LB broth with ampicillin. Plasmids were isolated using the Illustra plasmid kit (GE Healthcare, Carnaxide Portugal) and were sequenced (StabVida).

Relative levels of mRNAs for sea lamprey *ca18* and *ca19* genes were quantified by real-time RT-PCR analysis using SYBR green with an iQ5 Multicolor Real-Time PCR Detection System (Bio-Rad). See Ferreira-Martins ^70^ for details and supplemental Table 1 for *ca19* and *ca18* primer pairs. A melt curve was generated to confirm assay specificity and a dilution series prepared to check reaction efficiency. The *gapdh* was used as the housekeeping gene. The comparative cycle threshold (CT) method (2^-ΔΔCT^ method) based on CT values was used to analyze the expression levels of the genes of interest. Random resulting amplicons were run on 2% agarose TBE gel to confirm single amplified product with the expected size.

Tissue distributions of *ca18* and *ca19* in ammocoetes and post-metamorphic sea lamprey were assessed by quantitative RT-PCR (as described above). Blood contamination in the tissue samples was assessed using specific primers designed for sea lamprey hemoglobins that correspond to the major aHb component of this species, *hba2a* (=*aHb2a*) mRNA sequence ^71^ and a putative larval hemoglobin *hba9* (=*aHb9*). The transcript levels are indicated as relative mRNA levels per ng RNA. The ammocoete gill tissue was assigned a value of 1.0.

**Two-dimensional electrophoresis (2DE), MALDI-TOF/TOF analysis, protein identification and modeling.**

Since both cytosolic carbonic anhydrase isoforms are expressed in RBCs and due to the ease of blood collection we performed proteomic analysis using RBCs. RBC samples (150 µL) from post-metamorphic juveniles and ammocoetes were prepared for two-dimensional electrophoresis as described in Campos and co-workers ^67^. The 2DE gels were stained with Coomassie Blue Colloidal ^72^ or transferred to nitrocellulose membranes for 2-D western blotting as previously described in the immunoblotting methods section. Protein spots of interest identified through 2-D gel carbonic anhydrase probed western blots were excised from gels followed by in-gel digestion using the protease trypsin ^73^. For protein identification samples were analyzed using a 4700 Proteomics Analyzer MALDI-TOF/TOF (AB SCIEX, Foster City, CA, USA) as described ^67^. Peptide mass fingerprint (PMF) data was collected in positive MS reflector mode in the range of 700–4000 (m/z) and was calibrated internally using trypsin autolysis peaks. Several of the highest intensity and/or relevant tryptic peaks were selected for MS/MS analysis. Both MS and MS/MS spectra were analyzed using the software GPS Explorer (Version 3.6; ABSCIEX), against a locally stored copy of the UniProt protein sequence database (release 2011_12) using the Mascot search engine (Version 2.1.04) ^67^. Novel Ca19 isoform sequence was manually added to the database. The search included peaks with a signal-to-noise ratio greater than 10 and allowed for up to two missed trypsin cleavage sites. To be considered a match, a confidence interval (CI) of at least of 99%, calculated by AB SCIEX GPS Explorer software, was required.

*Petromyzon marinus* and *O. mykiss* carbonic anhydrase sequences were submitted to SwissModel (<http://swissmodel.expasy.org/>) for homology modelling ^74–76^, crystal structure of human CA2 (2CBA) was uploaded and used as a template. The resulting homology models were submitted to ModEval Model Evaluation Server available at <https://modbase.compbio.ucsf.edu/evaluation/> to estimate model quality, both models rendered as accurate. Homology model electrostatic potentials were calculated in PDB2PQR web server ^77^ (<http://www.poissonboltzmann.org/>). Force field AMBER was selected and pKa’s were calculated by PROPKA and assigned using pH 7. Electrostatic potential of the homology models were visualized online in APBS web solver.

**Supplemental Table 1**. Sets of primers used for RT-PCR, Real-time RT-PCR and RACE-PCR.

| **ID** | **Sequence 5’–3’** | **Reference** | **GenBank accession no/ ensembl.org** |
| --- | --- | --- | --- |
| *zfCA_F1* | CAG TTC CAT TTC CAT TGG GG |  | BC065611.1 |
| *zfCA_R1* | CAG AGG AGG GGT GGT CAG |  | BC065611.1 |
| *PmCA_F1* | ATA ACG CAG GGT TGC AGA AGG TGA C |  |  |
| *PmCA_F2* | GCT TTG ACG AGG CGA AAG ACA AGA G |  |  |
| *PmCA_R1* | GGA GAA CCG AGG GGT CGT AGT TCT T |  |  |
| *PmCA_R2* | GTC ACC TTC TGC AAC CCT GCG TTA T |  |  |
| *Pm_CA19-F* | ATA ACG CAG GGT TGC AGA AG |  |  |
| *Pm_CA19-R* | CTT TCA GTG AGC GGA ATG C |  |  |
| *Pm_CA18_F* | GCT GAA GCA GTT CCA CTT CC | 16 | DQ157849 |
| *Pm_CA18-R* | CCC TTG CTC CTG ATG ATG TT | 16 | DQ157849 |
| *Pm_aHb2a–F* | CAT GGA TGA CAC CGA GAA GA | 71 | AF248645 |
| *Pm_aHb2a-R* | GAC CTG AGC AGG ATG CAA AT | 71 | AF248645 |
| *Pm_aHb9-F* | AGA AGC ACG CTC AGG AGT TC |  | ENSPMAG00000008540 |
| *Pm_aHb9–R* | AGA GCA GCT GTT CGT TGT CA |  | ENSPMAG00000008540 |
| *Pm_gapdh-F* | TGC AAA GCA CGT CAT CAT CTC | 78 | AY578058 |
| *Pm_gapdh-R* | TTC TCG TGG TTT ACT CCC ATC A | 78 | AY578058 |
| *Pm_18S-F* | GTA GTT GGT GGA GCG ATT TGT CT | 79 | M97575 |
| *Pm_18S-R* | GGC CGC GTAG CTA GTT AGC A | 79 | M97575 |

**Supplemental Table2**. RT-PCR and RACE-PCR cycling profiles: zebrafish cytosolic carbonic anhydrase, *zfCA* and ; novel cytosolic carbonic anhydrase 19 isoform, *ca19.*

|  |  | *zfCA* | *Ca19* |
| --- | --- | --- | --- |
| Reaction type |  | RT-PCR | RACE |
| Denaturation and hot start |  | 94°C | 98°C |
|  |  | 30 s | 10s |
| Denature |  | 94°C | 98°C |
|  |  | 30 s | 1s |
| Anneal |  | 68°C | 68°C |
|  |  | 60 s | 5s |
| Extend |  | 72°C | 72°C |
|  |  | 90 s | 30s |
| Repeat cycles |  | 35 | 45 |
| Final extension |  | 72°C | 72°C |
|  |  | 10 min | 1min |

**Supplemental Table 3**. Real time RT-PCR conditions using iQ SYBR green supermix. Cytosolic carbonic anhydrase-18 , *ca18* ^16^; novel cytosolic carbonic anhydrase-19 isoform, *ca19*; putative larval hemoglobin, *hba9* ^71^; hemoglobin PMII, *hba2a* ^71^; Glyceraldehyde 3-phosphate dehydrogenase, *gapdh*; 18 subunit ribosomal RNA, *18S.*

|  | *ca18* | *Ca19* | *hba9* | *hba2a* | *gapdh* | *18s* |
| --- | --- | --- | --- | --- | --- | --- |
| Denaturation and hot start | 94°C | 94°C | 94°C | 94°C | 94°C | 94°C |
|  | 3.5 min | 3.5 min | 3.5 min | 3.5 min | 3.5 min | 3.5 min |
| Denaturation | 94°C | 94°C | 94°C | 94°C | 94°C | 94°C |
|  | 30 s | 30 s | 30 s | 30 s | 30 s | 30 s |
| Annealing | 60°C | 65°C | 60°C | 60°C | 60°C | 60°C |
|  | 30 s | 30 s | 30 s | 30 s | 30 s | 30 s |
| Extend | 72°C | 72°C | 72°C | 72°C | 72°C | 72°C |
|  | 30 s | 30 s | 30 s | 30 s | 30 s | 30 s |
| Repeat cycles | 40 | 40 | 40 | 40 | 40 | 40 |
|  |  |  |  |  |  |  |
| Melt Curve Analysis: 60°C to 95°C with 1°C increments for30 s each | | | | | | |

**Supplemental Table 4**. Sequence ID and accession numbers used for phylogenetic study.

| **Species** | **Gene** | **Accession** |
| --- | --- | --- |
| *H. sapiens* | CA13 | NP_940986 |
|  | CA1 | NP_001729 |
|  | CA3 | NP_005172 |
|  | CA2 | NP_000058 |
|  | CA7 | NP_005173 |
|  | CA5B | NP_009151 |
|  | CA5A | NP_001730 |
|  |  |  |
| *M. domestica* | CA13 | XP_001366749.3 |
|  | CA1 | NP_001028142.1 |
|  | CA3 | XP_001366645.1 |
|  | CA2 | XP_001376657.2 |
|  | CA5A | XP_007477337.1 |
|  | CA5B | XP_007500931.1 |
|  | CA7 | XP_001364411 |
|  |  |  |
| *G. gallus* | CA13 | XP_003640859 |
|  | CA3a | NP_001264339 |
|  | CA3b | NP_001264340 |
|  | CA2 | NP_990648 |
|  | CA7 | XP_414152 |
|  | CA5 | XP_414195 |
|  |  |  |
| *M. nubicus* | CA1 | XP_008940663.1 |
|  |  |  |
| *A. carolinensis* | CA3 | XP_003219614.1 |
|  |  |  |
| *P. sinensis* | CA1 | XP_006113045.1 |
|  | CA2 | XP_014437144.1 |
|  | CA7 | XP_006130611.1 |
|  | CA5 | XP_006118785.1 |
|  |  |  |
| *X. tropicalis* | Ca13 | NP_001072448 |
|  | Ca1 | XP_002939198 |
|  | Ca3 | XP_002939197 |
|  | Ca2b | NP_001015729 |
|  | Ca2a | NP_001072785 |
|  | Ca7 | AAI21633 |
|  | Ca5 | NP_001039155 |
|  |  |  |
| *L. chalumnae* | Ca17 (cahz) | XP_006010309 |
|  | Ca7 | XP_006004096 |
|  | Ca5 | XP_005989205 |
|  |  |  |
| *L. osseus* | Ca17 (Ca2-like) | AAM94169 |
|  |  |  |
| *L. oculatus* | Ca7 | XP_006641282 |
|  | Ca5 | XP_006641382.1 |
|  |  |  |
| *D. rerio* | Ca17b (Cahz) | NP_571185 |
|  | Ca17a (Ca2) | NP_954685 |
|  | Ca5 | NP_001104671 |
|  | Ca7 | AAH49309 |
|  |  |  |
| *O. latipes* | Ca17b (Cahz) | XP_011484743 |
|  | Ca17a (Ca2) | XP_004081218 |
|  | Ca5 | XP_004069790 |
|  |  |  |
| *G. morhua* | Ca17a (Ca2) | ENSGMOG00000015729 |
|  | Ca17b (Cahz) | ENSGMOG00000015697 |
|  | Ca5 | ENSGMOG00000004153 |
|  | Ca7 | ENSGMOG00000001295 |
|  |  |  |
| *G. aculeatus* | Ca5 | [ENSGACP00000004208](http://www.ensembl.org/Gasterosteus_aculeatus/Transcript/Sequence_Protein?db=core;g=ENSGACG00000003213;t=ENSGACT00000004222) |
|  |  |  |
| *P. marinus* | Ca19 | KT030772 |
|  | Ca18 | AAZ83742 |
|  | Ca5 | ENSPMAP00000010279 |
|  |  |  |
| *L. japonicum* | Ca19 | [JL12788](http://blast.imcb.a-star.edu.sg/cgi-bin/scripts/request_scaff.pl?db=jlamp_proteins&seqid=JL12788) |
|  | Ca18 | JL10053 |
|  |  |  |
| *S. canicula* | Ca5 | ctg95805 |
|  |  |  |
| *C. milii* | Ca17 (Ca2-like) | AFK10663 |
|  | Ca5 | XP_007887550 |
|  | Ca7 | XP_007906064 |
|  |  |  |
| *L. erinacea* | Ca5 | ctg14021 |
|  | Ca17 (Ca2-like) | ctg70321 |
|  |  |  |
| *B. floridae* | Ca | XP_002594303 |
|  |  |  |
| *D. melanogaster* | CA | NP_523561 |

**Supplemental Table 5**. Protein identification by MALDI-TOF/TOF analysis. Identification scores obtained with the paragon algorithm from the protein pilot program (*). n.o. = no observation.

| **Spot #** | **Protein name** | **Identified**  **protein and NCBI**  **database reference**  **(1)** | **Organism** | **Molecular mass (kDa) (2)** | **pI (3)** | **Identification parameters** | | | | | | |
| --- | --- | --- | --- | --- | --- | --- | --- | --- | --- | --- | --- | --- |
|  |  |  |  |  |  | **Ident. score (4)** | **Total ion score (5)** | **Matched fragmented peptides (6)** | **sequence coverage % (7)** | **M+H^+^**  **(8)** | **Peptide sequence**  **(9)** | **Ion score**  **(10)** |
| Ca18 i | Carbonic anhydrase | tr|Q3Y546|Q3Y546  _PETMA | *Petromyzon marinus* | 29 | 5.47 | 399 | 327 | 3 | 100 | 1401.7195  1620.8519  1755.8813 | CVLSGGPLPNPYK  VDFLDYDPSVLLPK  TYSAELHLVHWNSAK | 90  92  145 |
|  |  |  |  |  |  |  |  |  |  |  |  |  |
|  |  |  |  |  |  |  |  |  |  |  |  |  |
| Ca18 ii | Carbonic anhydrase | tr|Q3Y546|Q3Y546  _PETMA | *Petromyzon marinus* | 29 | 5.47 | 249 | 224 | 4 | 100 | 837.41  1044.6049  1128.5684  1401.7195 | SFAEAANK  VTDTLNIIR  YKSFAEAANK  CVLSGGPLPNPYK | 73  29  89  33 |
|  |  |  |  |  |  |  |  |  |  |  |  |  |
| Ca19 i | Pma ammocoete carbonic anhydrase | n.o. | *Petromyzon marinus* | 27.6 | 6.23 | 362 | 267 | 3 | 100 | 834.4468  1430.66  1725.8707 | EQLAAFR  QFHFHWGASDAK  SYSAELHLVHWNAAK | 47  104  116 |
|  |  |  |  |  |  |  |  |  |  |  |  |  |
| Ca19 ii | Pma ammocoete carbonic anhydrase | n.o. | *Petromyzon marinus* | 27.6 | 6.23 | 468 | 335 | 2 | 100 | 834.4468  1430.66 | EQLAAFR  QFHFHWGASDAK | 48  86 |
|  |  |  |  |  |  |  |  |  |  |  |  |  |
| Ca19 iii | Pma ammocoete carbonic anhydrase | n.o. | *Petromyzon marinus* | 27.6 | 6.23 | 440 | 333 | 3 | 100 | 834.4468  1430.66  1472.8042 | EQLAAFR  QFHFHWGASDAK  LVCNFRPTQPLK | 52  75  62 |
|  |  |  |  |  |  |  |  |  |  |  |  |  |
| Ca19 iv | Pma ammocoete carbonic anhydrase | n.o. | *Petromyzon marinus* | 27.6 | 6.23 | 463 | 343 | 4 | 100 | 834.4468  1430.66  1472.8042  1725.8707 | EQLAAFR  QFHFHWGASDAK  LVCNFRPTQPLK  SYSAELHLVHWNAAK | 45  108  46  144 |
|  |  |  |  |  |  |  |  |  |  |  |  |  |
| Ca19 v | Pma ammocoete carbonic anhydrase | n.o. | *Petromyzon marinus* | 27.6 | 6.23 | 457 | 337 | 4 | 100 | 834.4468  1430.66  1472.8042  1725.8707 | EQLAAFR  QFHFHWGASDAK  LVCNFRPTQPLK  SYSAELHLVHWNAAK | 47  86  60  144 |
| Ca19 vi | Pma ammocoete carbonic anhydrase | n.o. | *Petromyzon marinus* | 27.6 | 6.23 | 458 | 338 | 3 | 100 | 834.4468 1430.66  1472.8042 | EQLAAFR  QFHFHWGASDAK  LVCNFRPTQPLK | 44  103  46 |

1) Accession number of the identified protein from the NCBI database. 2) Protein theoretical molecular mass. 3) Protein theoretical isoelectric point. 4) Identification scores obtained with the algorithms Mowse or Paragon. Minimum scores were 78 and 2 for the Mowse and Paragon algorithms respectively corresponding to a P<0.05. 5) The sum of all individual ion scores of the fragmented ions. 6) Different peptides matching the sequence of the identified protein. 7) Percentage of the identified protein sequence covered by the matched peptides. 8) Monoisotopic masses of the fragmented peptides. 9) Sequence of the fragmented peptides. 10) Individual ion scores from the Mowse algorithm respecting the identification of peptide sequences. Values obtained correspond to a P<0.05.

**Supplemental Table 6A.** Electrostatic potential of carbonic anhydrase from teleost fishes corresponding to the tissue-cell type (TC: Ca17 = Ca2-like a = Cac) or red blood cell (RBC) type (Ca17b = Ca2-like b = Cahz). **(B)** Electrostatic potential distribution mapped on the Gaussian-type surface calculated by APBS and visualized with PyMol for trout and lamprey carbonic anhydrases. The potentials range from -5.0kT per proton charge (blue) to 5.0kT per proton charge (red). The structures are graphically depicted looking down the active-site cleft (upper) and in a 180º rotated view (lower).

A

| **Spp** | **CA** | **ID** | **Total protein charge** | **Comment** |
| --- | --- | --- | --- | --- |
| *Danio rerio* | Ca17 b | Q92051 | 0.00 | RBC; FW |
| *Oreochromis niloticus* | Ca17 b | ENSONIP00000008038 | 1.00 | RBC; FW |
| *Oryzias latipes* | Ca17 b | ENSORLP00000010477 | 1.00 | RBC; FW |
| *Poecilia formosa* | Ca17 b | ENSPFOP00000002771 | 1.00 | RBC; FW |
| *Xiphophorus maculatus* | Ca17 b | ENSXMAP00000001090 | 1.00 | RBC; FW |
| *Danio rerio* | Ca17 a | Q6PFU7 | 5.00 | RBC; FW |
| *Gasterosteus aculeatus* | Ca17 b | ENSGACP00000019535 | 1.00 | RBC; FW-EUR |
| *Gadus morhua* | Ca17 b | ENSGMOP00000016838 | 1.00 | RBC; SW |
| *Takifugu rubripes* | Ca17 b | ENSTRUP00000020144 | 1.00 | RBC; SW |
| *Tetraodon nigrovidis* | Ca17 b | ENSTNIP00000008780 | 2.00 | RBC; SW-EUR |
| *Petromyzon marinus* | Ca18 |  | -8.00 | RBC/TC; FW-EUR |
| *Petromyzon marinus* | Ca19 |  | -2.00 | RBC/TC; FW |
| *Onchorhynchus mykiss* | Ca17 b | Q7T2K6 | 1.00 | RBC/TC; FW |
| *Gadus morhua* | Ca17 a | ENSGMOP00000016873 | -7.00 | TC; SW |
| *Takifugu rubripes* | Ca17 a | ENSTRUP00000029310 | -1.00 | TC; SW |
| *Gasterosteus aculeatus* | Ca17 a | ENSGACP00000006664 | -2.00 | TC; EUR |
| *Poecilia formosa* | Ca17 a | ENSPFOP00000009383 | -12.00 | TC; FW |
| *Xiphophorus maculatus* | Ca17 a | ENSXMAP00000008413 | -8.00 | TC; FW |
| *Oreochromis niloticus* | Ca17 a | ENSONIP00000008037 | -2.00 | TC; FW-EUR |
| *Oryzias latipes* | Ca17 a | ENSORLP00000015962 | 0.00 | TC; FW |
| *Tetraodon nigrovidis* | Ca17 a | ENSTNIP00000016652 | -5.00 | TC; SW |
| *Onchorhynchus mykiss* | Ca17 a | Q68YC2 | -6.00 | TC; EUR |
| *Chionodraco hamatus* | Ca17 a | P83299 | -7.00e | TC; SW |
|  |  |  |  |  |


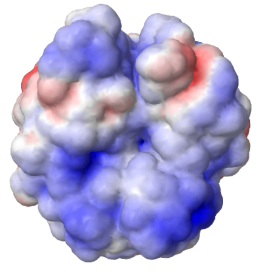

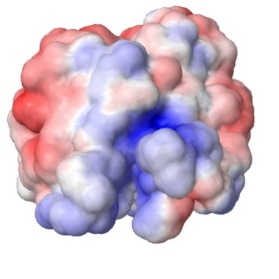


Omyk Ca17b

Q7T2K6

Total charge 1.0e


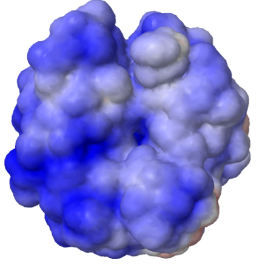

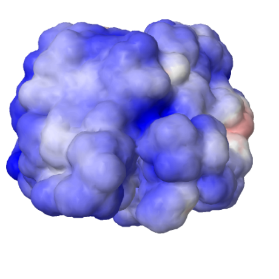


Omyk Ca17a

Q68YC2

Total charge -6.0e


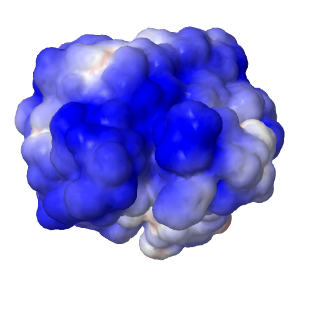

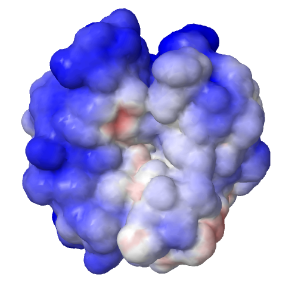


Pmar Ca18

Total charge -8.0e


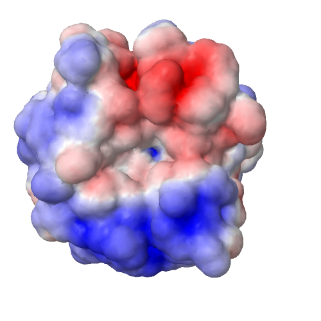

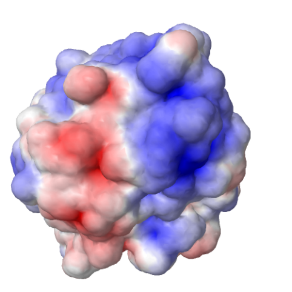


Pmar Ca19

Total charge -2.0e

B

**Supplemental Table 7**. Predicted phosphorylation analysis using ProMoST for proteins Ca18 and Ca19. Protein mass in expressed in Daltons (Da); pI, isoelectric point; phosST, phosphorylation serine-threonine; phosY, phosphorylation tyrosine.

|  | **Protein** | **Average mass (Da)** | **pI** | **phosST** | **phosY** |
| --- | --- | --- | --- | --- | --- |
|  | Ca18 | 28795.28 | 5.51 | 0 | 0 |
|  | Ca18 | 28795.28 | 5.39 | 1 | 0 |
|  | Ca18 | 28795.28 | 5.27 | 2 | 0 |
|  | Ca18 | 28795.28 | 5.17 | 3 | 0 |
|  | Ca18 | 28795.28 | 5.07 | 4 | 0 |
|  | Ca18 | 28795.28 | 4.98 | 5 | 0 |
|  | Ca18 | 28795.28 | 4.91 | 6 | 0 |
|  | Ca18 | 28795.28 | 4.83 | 7 | 0 |
|  | Ca18 | 28795.28 | 4.76 | 8 | 0 |
|  | Ca18 | 28795.28 | 4.70 | 9 | 0 |
|  | Ca18 | 28795.28 | 4.64 | 10 | 0 |
|  | Ca18 | 28795.28 | 5.39 | 0 | 1 |
|  | Ca18 | 28795.28 | 5.27 | 0 | 2 |
|  | Ca18 | 28795.28 | 5.17 | 0 | 3 |
|  | Ca18 | 28795.28 | 5.07 | 0 | 4 |
|  | Ca18 | 28795.28 | 4.98 | 0 | 5 |
|  | Ca18 | 28795.28 | 4.90 | 0 | 6 |
|  | Ca18 | 28795.28 | 4.83 | 0 | 7 |
|  | Ca18 | 28795.28 | 4.76 | 0 | 8 |
|  | Ca18 | 28795.28 | 4.70 | 0 | 9 |
|  | Ca18 | 28795.28 | 4.64 | 0 | 10 |
|  | Ca19 | 27456.60 | 6.51 | 0 | 0 |
|  | Ca19 | 27456.60 | 6.27 | 1 | 0 |
|  | Ca19 | 27456.60 | 6.08 | 2 | 0 |
|  | Ca19 | 27456.60 | 5.91 | 3 | 0 |
|  | Ca19 | 27456.60 | 5.75 | 4 | 0 |
|  | Ca19 | 27456.60 | 5.59 | 5 | 0 |
|  | Ca19 | 27456.60 | 5.44 | 6 | 0 |
|  | Ca19 | 27456.60 | 5.30 | 7 | 0 |
|  | Ca19 | 27456.60 | 5.17 | 8 | 0 |
|  | Ca19 | 27456.60 | 5.05 | 9 | 0 |
|  | Ca19 | 27456.60 | 4.95 | 10 | 0 |
|  | Ca19 | 27456.60 | 6.27 | 0 | 1 |
|  | Ca19 | 27456.60 | 6.08 | 0 | 2 |
|  | Ca19 | 27456.60 | 5.91 | 0 | 3 |
|  | Ca19 | 27456.60 | 5.75 | 0 | 4 |
|  | Ca19 | 27456.60 | 5.59 | 0 | 5 |
|  | Ca19 | 27456.60 | 5.44 | 0 | 6 |
|  | Ca19 | 27456.60 | 5.30 | 0 | 7 |
|  | Ca19 | 27456.60 | 5.17 | 0 | 8 |
|  | Ca19 | 27456.60 | 5.05 | 0 | 9 |

**Supplemental Table 8**. Predicted phosphorylation site analysis using NetPhos 3.1 Server for Ca18 and Ca19. aa, amino acid; cdc2, cell division control 2; CKI, casein kinase 1; CKII, casein kinase 2; DNAPK, DNA-dependent protein kinase; EGFR, epidermal growth factor receptor kinase; INSR, insulin receptor tyrosine kinase; p38MAPK, P38 mitogen-activated protein kinase; PKA, protein kinase A; PKC, protein kinase C; PKG, protein kinase G; unsp, unspecific kinase. Predicted phosphorylated aa represented in context centered in the aa motif.

| **Sequence** | **aa position** | **aa** | **Context** | **Score** | **Kinase** |
| --- | --- | --- | --- | --- | --- |
| Ca18 | 30 | S | GERQSPIDI | 0.997 | unsp |
| Ca18 | 40 | T | PGEATYDAT | 0.869 | unsp |
| Ca18 | 41 | Y | GEATYDATL | 0.531 | unsp |
| Ca18 | 44 | T | TYDATLKPL | 0.734 | PKC |
| Ca18 | 44 | T | TYDATLKPL | 0.593 | unsp |
| Ca18 | 49 | S | LKPLSVIYD | 0.961 | unsp |
| Ca18 | 49 | S | LKPLSVIYD | 0.558 | PKC |
| Ca18 | 49 | S | LKPLSVIYD | 0.528 | PKA |
| Ca18 | 56 | S | YDPASALSM | 0.514 | cdc2 |
| Ca18 | 59 | S | ASALSMGNN | 0.905 | unsp |
| Ca18 | 59 | S | ASALSMGNN | 0.529 | cdc2 |
| Ca18 | 59 | S | ASALSMGNN | 0.514 | CKI |
| Ca18 | 66 | S | NNGHSFSVE | 0.532 | cdc2 |
| Ca18 | 68 | S | GHSFSVEYD | 0.869 | unsp |
| Ca18 | 71 | Y | FSVEYDDSG | 0.961 | unsp |
| Ca18 | 81 | S | KCVLSGGPL | 0.622 | PKA |
| Ca18 | 104 | S | AADGSGSEH | 0.969 | unsp |
| Ca18 | 104 | S | AADGSGSEH | 0.603 | CKII |
| Ca18 | 109 | T | GSEHTVAGK | 0.573 | PKC |
| Ca18 | 114 | T | VAGKTYSAE | 0.606 | PKC |
| Ca18 | 126 | S | VHWNSAKYK | 0.718 | PKC |
| Ca18 | 129 | Y | NSAKYKSFA | 0.900 | unsp |
| Ca18 | 131 | S | AKYKSFAEA | 0.963 | unsp |
| Ca18 | 131 | S | AKYKSFAEA | 0.517 | PKG |
| Ca18 | 170 | S | NIIRSKGAK | 0.769 | PKC |
| Ca18 | 198 | S | TYLGSLTTP | 0.586 | PKC |
| Ca18 | 198 | S | TYLGSLTTP | 0.527 | PKA |
| Ca18 | 201 | T | GSLTTPPLF | 0.584 | p38MAPK |
| Ca18 | 209 | T | FESVTWIVF | 0.655 | PKC |
| Ca18 | 220 | S | PIPASKEQL | 0.828 | unsp |
| Ca18 | 233 | T | ELLFTCEGD | 0.509 | CKII |
| Ca18 | 233 | T | ELLFTCEGD | 0.502 | CKI |
| Ca18 | 238 | S | CEGDSENCM | 0.705 | unsp |
| Ca18 | 238 | S | CEGDSENCM | 0.532 | CKII |
| Ca18 | 246 | Y | MVDNYRPPQ | 0.501 | EGFR |
| Ca18 | 256 | T | LGGRTVRAS | 0.610 | PKC |
| Ca18 | 256 | T | LGGRTVRAS | 0.539 | unsp |
| Ca18 | 260 | S | TVRASFQ-- | 0.838 | unsp |
| Ca18 | 260 | S | TVRASFQ-- | 0.540 | PKA |
| Ca19 | 7 | Y | EHWGYGSEN | 0.553 | unsp |
| Ca19 | 9 | S | WGYGSENGP | 0.526 | CKII |
| Ca19 | 29 | S | GSRQSPIDI | 0.998 | unsp |
| Ca19 | 29 | S | GSRQSPIDI | 0.511 | CKI |
| Ca19 | 48 | S | LGALSVSYS | 0.604 | PKC |
| Ca19 | 50 | S | ALSVSYSGA | 0.937 | unsp |
| Ca19 | 50 | S | ALSVSYSGA | 0.563 | cdc2 |
| Ca19 | 52 | S | SVSYSGADA | 0.975 | unsp |
| Ca19 | 58 | S | ADAKSISNS | 0.956 | unsp |
| Ca19 | 58 | S | ADAKSISNS | 0.566 | cdc2 |
| Ca19 | 60 | S | AKSISNSGH | 0.678 | unsp |
| Ca19 | 60 | S | AKSISNSGH | 0.563 | cdc2 |
| Ca19 | 62 | S | SISNSGHSF | 0.682 | PKC |
| Ca19 | 62 | S | SISNSGHSF | 0.680 | unsp |
| Ca19 | 65 | S | NSGHSFSVD | 0.951 | unsp |
| Ca19 | 67 | S | GHSFSVDYD | 0.595 | unsp |
| Ca19 | 67 | S | GHSFSVDYD | 0.501 | CKII |
| Ca19 | 70 | Y | FSVDYDDSG | 0.975 | unsp |
| Ca19 | 77 | S | SGDSSVLSG | 0.728 | unsp |
| Ca19 | 80 | S | SSVLSGGPL | 0.839 | unsp |
| Ca19 | 100 | S | HWGASDAKG | 0.591 | PKC |
| Ca19 | 108 | T | GSEHTVDGK | 0.549 | PKC |
| Ca19 | 113 | S | VDGKSYSAE | 0.984 | unsp |
| Ca19 | 113 | S | VDGKSYSAE | 0.515 | cdc2 |
| Ca19 | 114 | Y | DGKSYSAEL | 0.502 | INSR |
| Ca19 | 115 | S | GKSYSAELH | 0.666 | unsp |
| Ca19 | 128 | Y | NAAKYASFD | 0.849 | unsp |
| Ca19 | 130 | S | AKYASFDEA | 0.970 | unsp |
| Ca19 | 130 | S | AKYASFDEA | 0.582 | CKII |
| Ca19 | 138 | S | AKDKSDGLA | 0.672 | unsp |
| Ca19 | 138 | S | AKDKSDGLA | 0.540 | PKA |
| Ca19 | 161 | T | LQKVTDALN | 0.516 | CKII |
| Ca19 | 182 | S | NYDPSVLLP | 0.577 | PKC |
| Ca19 | 193 | S | LDFWSYLGS | 0.507 | cdc2 |
| Ca19 | 197 | S | SYLGSLTTP | 0.686 | PKC |
| Ca19 | 197 | S | SYLGSLTTP | 0.591 | PKA |
| Ca19 | 200 | T | GSLTTPPLN | 0.584 | p38MAPK |
| Ca19 | 206 | S | PLNESAIWI | 0.503 | PKG |
| Ca19 | 219 | S | PISASKEQL | 0.833 | unsp |
| Ca19 | 228 | S | AAFRSLKAS | 0.553 | PKC |
| Ca19 | 232 | S | SLKASDGSK | 0.846 | unsp |
| Ca19 | 232 | S | SLKASDGSK | 0.579 | PKC |
| Ca19 | 235 | S | ASDGSKLVC | 0.507 | cdc2 |
| Ca19 | 244 | T | NFRPTQPLK | 0.598 | DNAPK |
| Ca19 | 244 | T | NFRPTQPLK | 0.587 | PKC |
| Ca19 | 255 | S | VVQASFK-- | 0.935 | unsp |
| Ca19 | 255 | S | VVQASFK-- | 0.807 | PKC |

**Supplemental Figure 1**. Alignment of *P. marinus* (Pma) sequence obtained from ammocoete gill (Ca19) with published *P. marinus* Ca18 (GenBank, AAZ83742.1), *Equus caballus* (Eca) CA (GenBank, P00917.3), *Homo sapiens* (Hsa) CA2 (GenBank, AAA51908.1), and *Oncorhynchus mykiss* (Omy) Ca17a (GenBank, AAR99329.1) aa sequences using ClustalW (BioEdit 7.0.9.0). Predicted phosphorylation sites using NetPhos 3.1 Server for Ca18 (+) and Ca19 (*), respectively.

**10 20 30 40 50 60 70 80 90**

**....|....|....|....|....|....|....|....|....|....|....|....|....|....|....|....|....|....|**

**Pma Ca19 * * * * * * * * * * * * * ***

**Pma Ca18 + ++ + + + + + + + +**

**Pma Ca19 -MCEHWGYGSENGPEVWGKHFKNADGSRQSPIDIEPASAVYEAALGALSVSYSGADAKSISNSGHSFSVDYDDSGDSSVLSGGPLANPYK**

**Pma Ca18** **MSGH.....E....AE.H.D.QI.K.E.......Q.GE.T.D.T.KP...I.DP.S.L.MG.N......E.....EKC.......P....**

**Eca CA**  **MAHSD...D.P...ZE.V.LYPI.N.NN......KTSETKHDTS.KPF....DP.T..E.V.V....Q.KFE..DNR...KD...PGS.R**

**Hsa CA2 MSH-.....KH....H.H.D.PI.K.E....V..DTHT.K.DPS.KP.....DQ.TSLR.L.N..A.N.EF...Q.KA..K....DGT.R**

**Omy Ca17aMSH-A...APD...DK.CEG.PI.N.P.......V.GE.AFD...K..TLK.DPSTSID.L.N....Q.T.T.DN.N.T.T...ISGT.R**

**100 110 120 130 140 150 160 170 180**

**....|....|....|....|....|....|....|....|....|....|....|....|....|....|....|....|....|....|**

**Pma Ca19 * * *** * * * ***

**Pma Ca18 + + + + + + +**

**Pma Ca19 LKQFHFHWGASDAKGSEHTVDGKSYSAELHLVHWNAAKYASFDEAKDKSDGLAVLGAFVKVGANNAGLQKVTDALNAIADKGAKADFKNY**

**Pma Ca18 ..........A.GS......A..T...........S...K..A..AN.........V.LEA..E.P..K....T..I.RS....V..LD.**

**Eca CA**  **.V.......ST.DY........VK...........SS..S.....SSQA....I..VLM...EA.PK....L....EVKT..K..P...F**

**Hsa CA2** **.I.......SL.GQ.......K.K.A.........-T..GD.GK.VQQP.......I.L...SAKP.....V.V.DS.KT..KS...T.F**

**Omy Ca17a............DR......A.TK.A.........-T..P..GD.AS.......V.V.LQ..NE..N....L..FD..KA..KQTS.E.F**

**190 200 210 220 230 240 250 260**

**....|....|....|....|....|....|....|....|....|....|....|....|....|....|....|....|..**

**Pma Ca19 * * * * * * * * * * ***

**Pma Ca18 + + + + + + + + +**

**Pma Ca19 DPSVLLPKSLDFWSYLGSLTTPPLNESAIWIVLKDPISASKEQLAAFRSLKAS-DGSK---LVCNFRPTQPLKGRVVQASFK**

**Pma Ca18 .............T..........F..VT...F.E..P.......R..E.LFTCE.DSENCM.D.Y..P...G..T.R...Q**

**Eca CA**  **...S...S.P.Y.T.S....H...Y..VT...C.EN..I.SQ..SQ....LSNVE.G.AVPIQH.N..P......T.R.F.-**

**Hsa CA2 ..RG...E...Y.T.P........L.CVT.....E...V.S..VLK..K.NFNGE.EPEELM.D.W..A....N.QIK....**

**Omy Ca17a..TI.......Y.T.D........L..VT...C.ES..V.PA.MGK....LF.GE.EAACCM.D.Y..P......AIT.RS-**


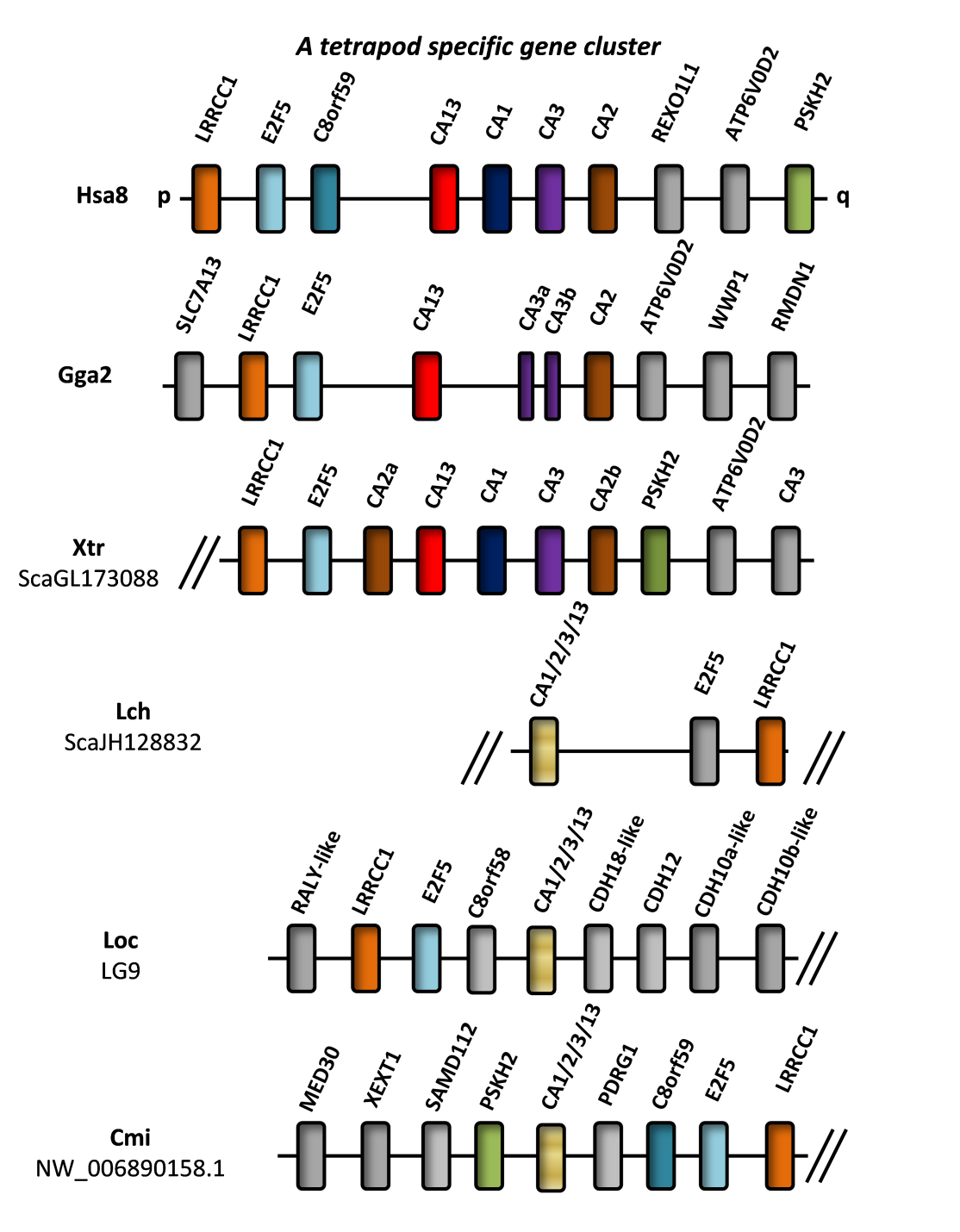


**Supplemental Figure 2**. Synteny maps of *CA* *loci*. Hsa – *H. sapiens*, Xtr- *X. tropicalis* and Lch- *L. chalumnae*.

Sca_GL481555

***CA5***

***HEATR3***

**Hsa16**

**50.1Mb**

8.3/5; 6.1;18

16.3;19.4

14.1

20.4

**A**

**B**

**C**

**D**

***CA5A****

**Hsa16 87Mb**

***CA7***

**Hsa16 66Mb**

***CA1/2/3/13***

**Hsa8 87Mb**

***ca19***

***c14orf119***

***ccne3***

**Ccne1-19(30)**

**Ccne2-8(95)**

**PmaCcne3**

**Hsa14 23Mb**

**Supplemental Figure 3**. Paralogy analysis of the *CA* *loci* in the human genome and their comparison to the *ca locus* composition in lamprey (see Putnam et al.^21^ for details of chromosome coordinates of linkage group 3); small grey circles denote genes whose human orthologues are either absent or map to a distinct linkage group.

**Supplemental Figure 4.** Transcript level expression of Glyceraldehyde-3-phosphate dehydrogenase (*gapdh*) ^78^ and Small subunit (18S) ribosomal RNA gene (*18s*) ^79^ in *P. marinus* ammocoete and post-metamorphic juvenile gill, red blood cells (RBC), kidney (Kid), anterior (AntInt) and posterior (PostInt) intestine determined by qPCR. Data was analyzed by two way ANOVA followed by SNK post-hoc test (N=4). Tissue expression levels are significantly different if they lack common letters. Significant differences between ammocoete and juvenile within a given tissue are indicated by an asterisk.

**Supplemental Figure 5.** Fold change of *ca18* determined by quantitative real-time PCR (mean ± S.E.M.) in *Petromyzon marinus* post-metamorphic juveniles acclimated to dH_2_O (N=3), fresh water (N=5), and salinities of 10‰ (N=2), 20‰ (N=6) and 30‰ (N=6). Bars with like characters are not significantly different from each other.


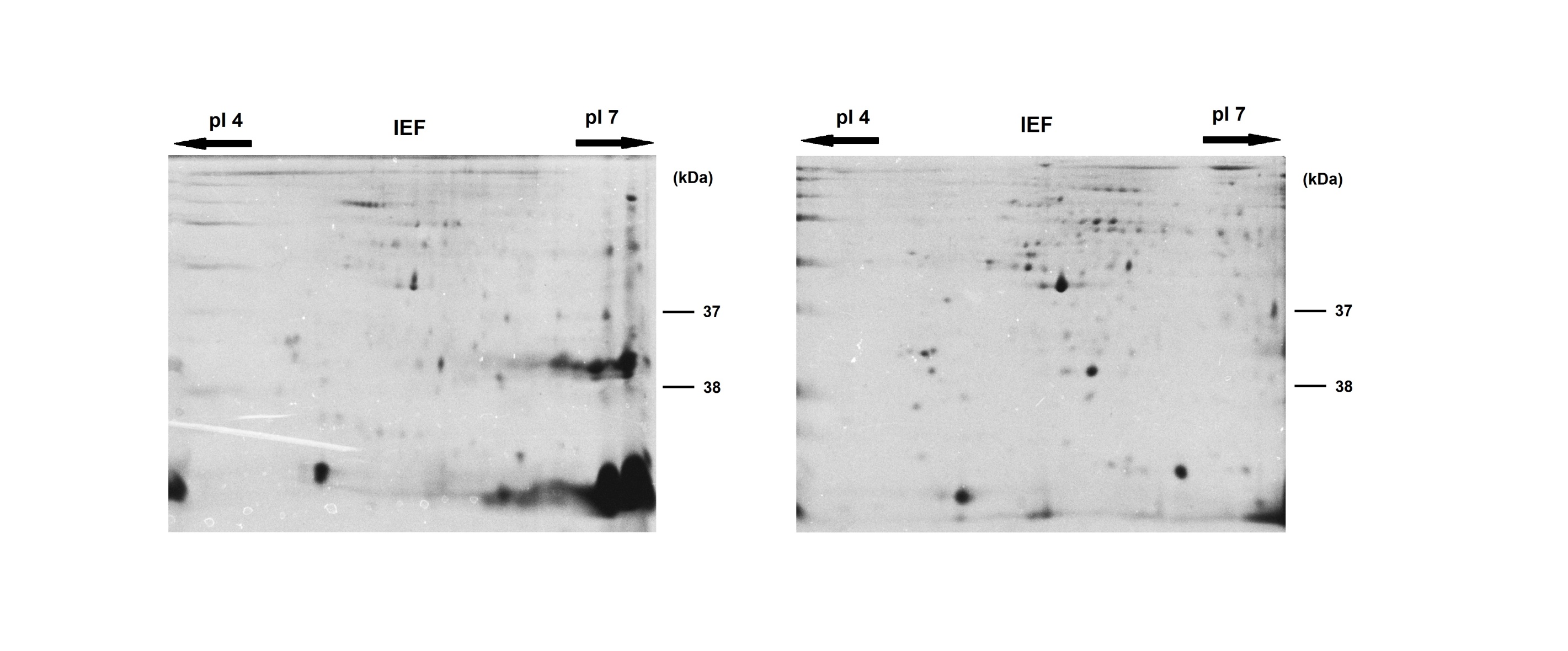

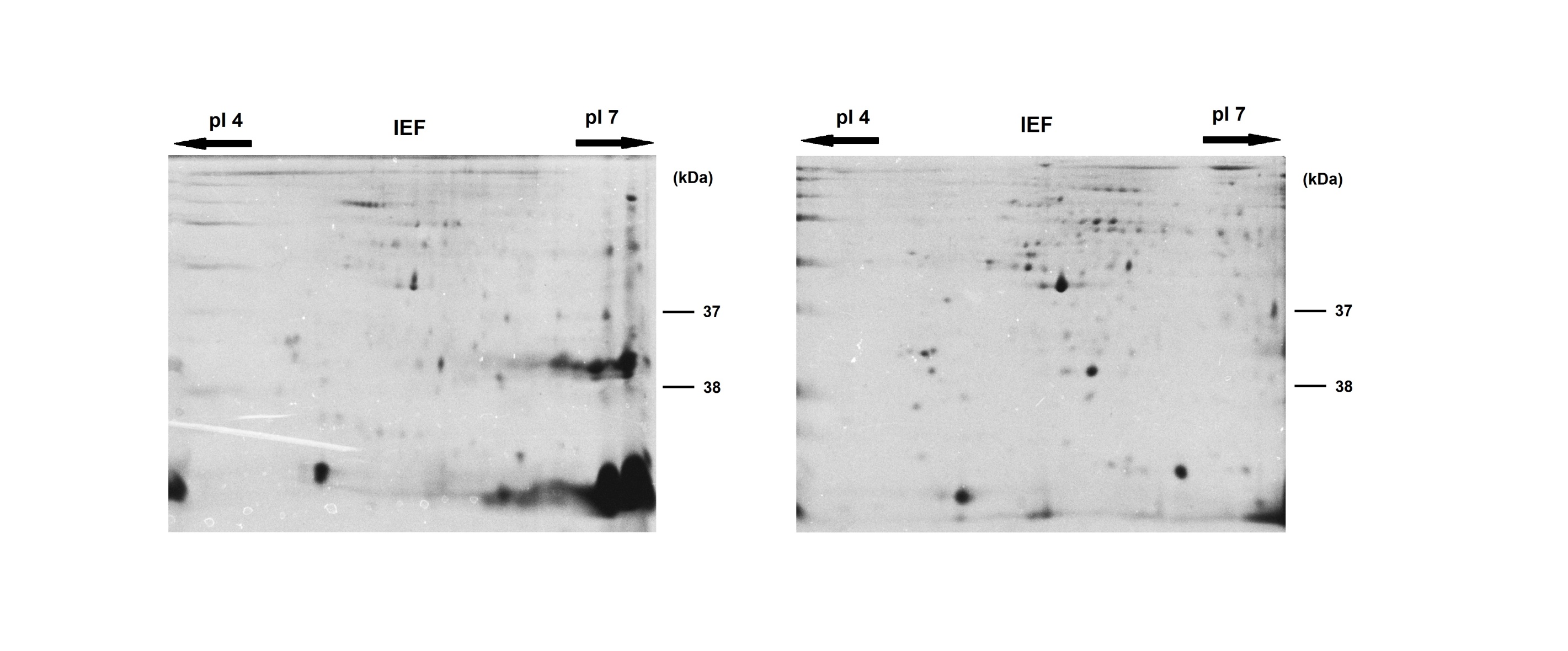

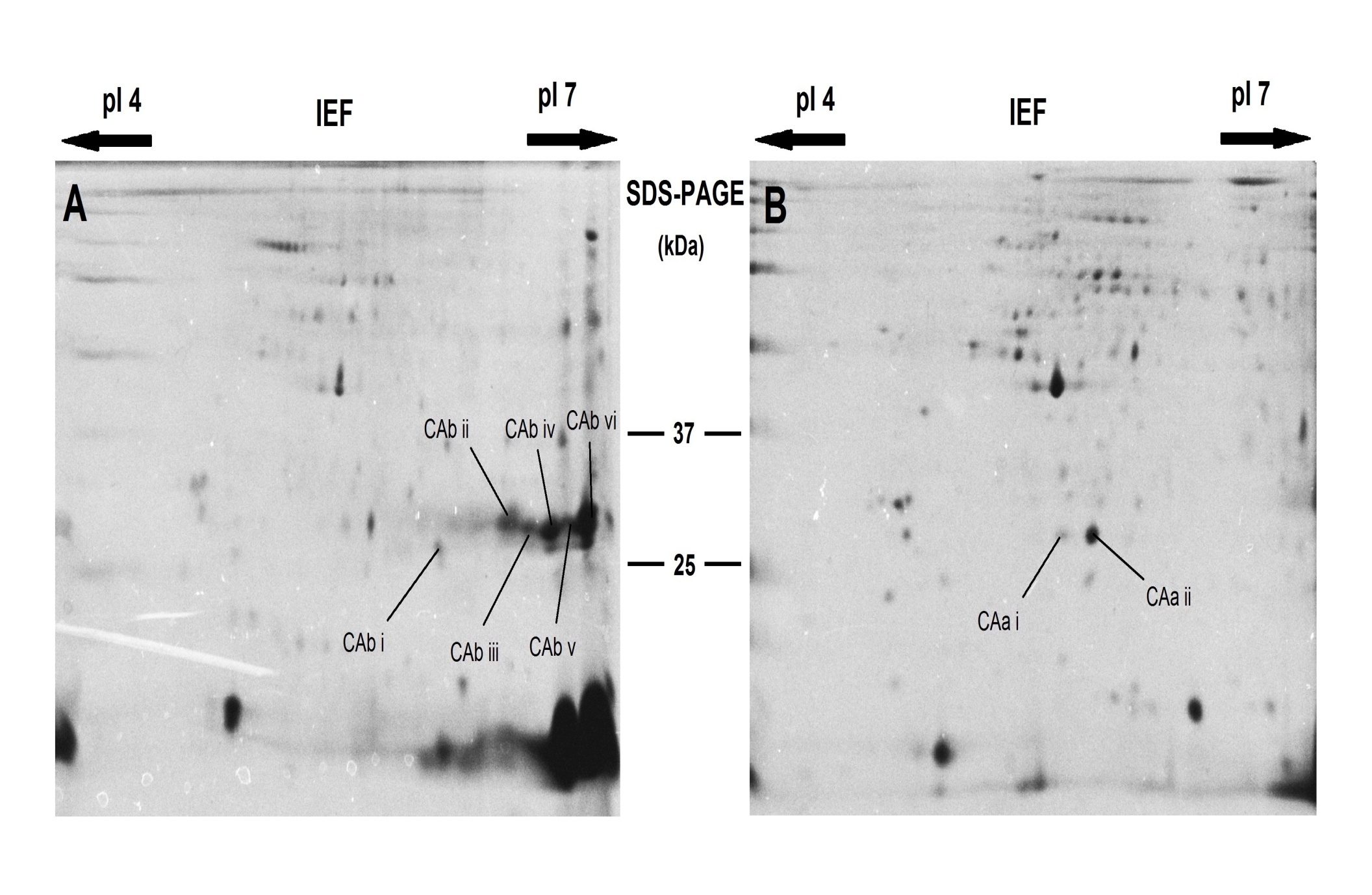

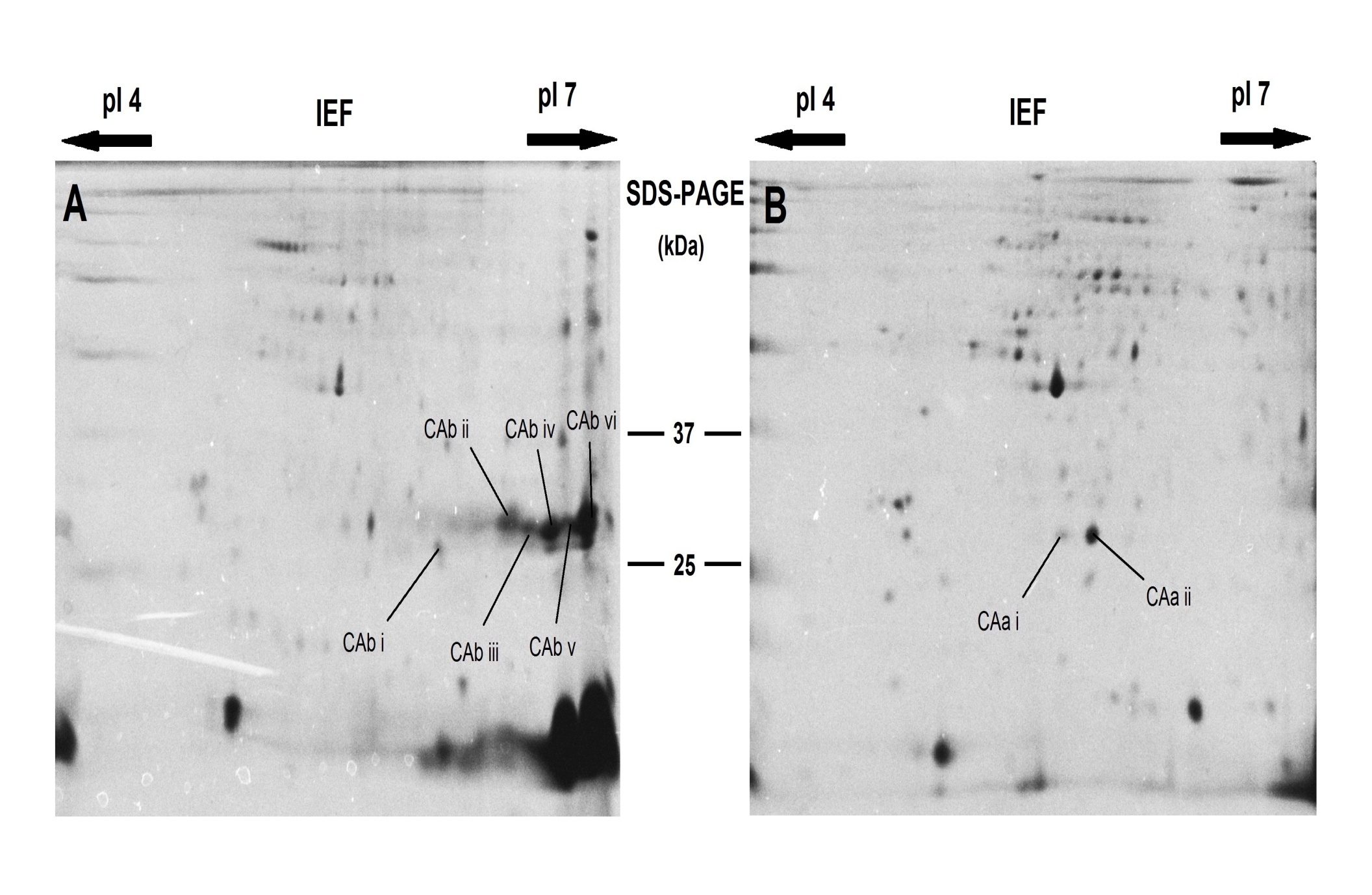

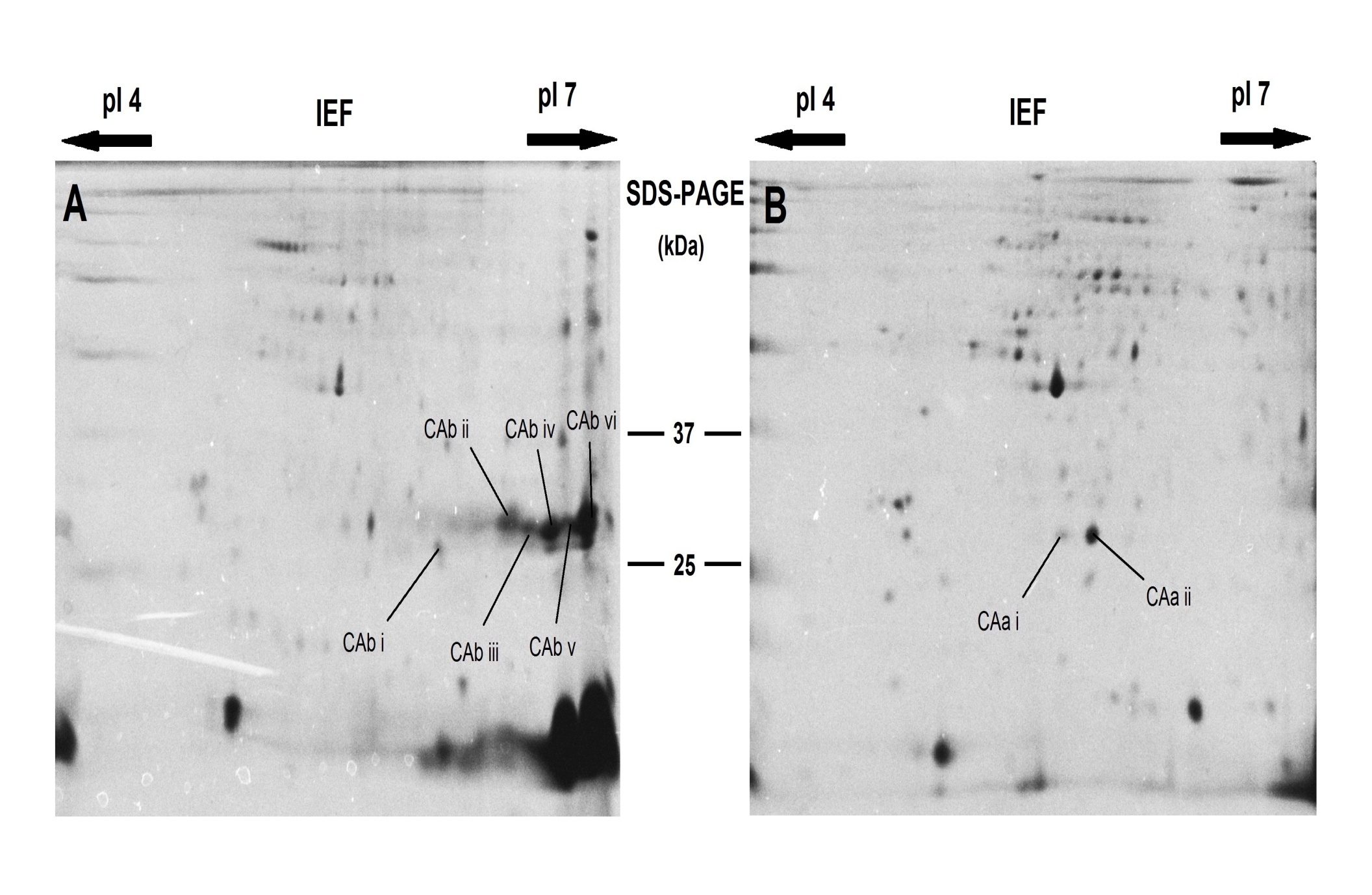

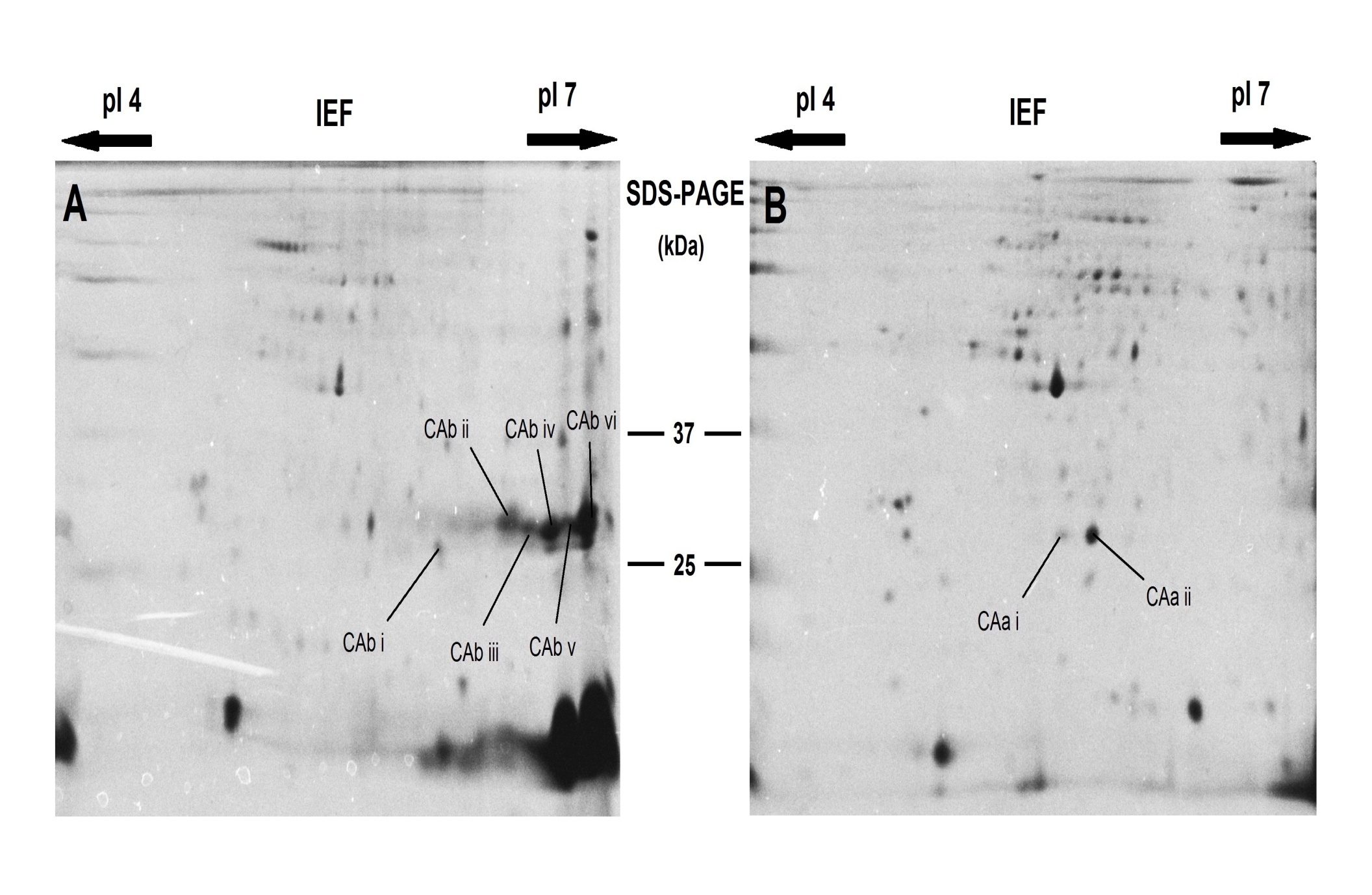


**IEF**

**IEF**

**37**

**25**

**SDS-PAGE**

**(kDa)**

**pI 4**

**pI 7**

**pI 4**

**pI 7**

Ca19 i

Ca19 ii

Ca19 iii

Ca19 iv

Ca19 v

Ca19 vi

Ca18 i

Ca18 ii

**A**

**B**

Ca18 i

Ca18 ii

**Supplemental Figure 6**. 2-DE gels of ammocoete (A) and juvenile (B) red blood cell samples. The gels were each loaded with 150 µg protein. Proteins were first separated by IEF in a pH gradient from 4 to 7 and further by SDS-PAGE (12%T). Gels were stained by Coomassie Blue Colloidal as described by Consoli and Damerval ^80^. Spots identified as carbonic anhydrase a (Ca18) and carbonic anhydrase b (Ca19) by MS/MS and database search are indicated.


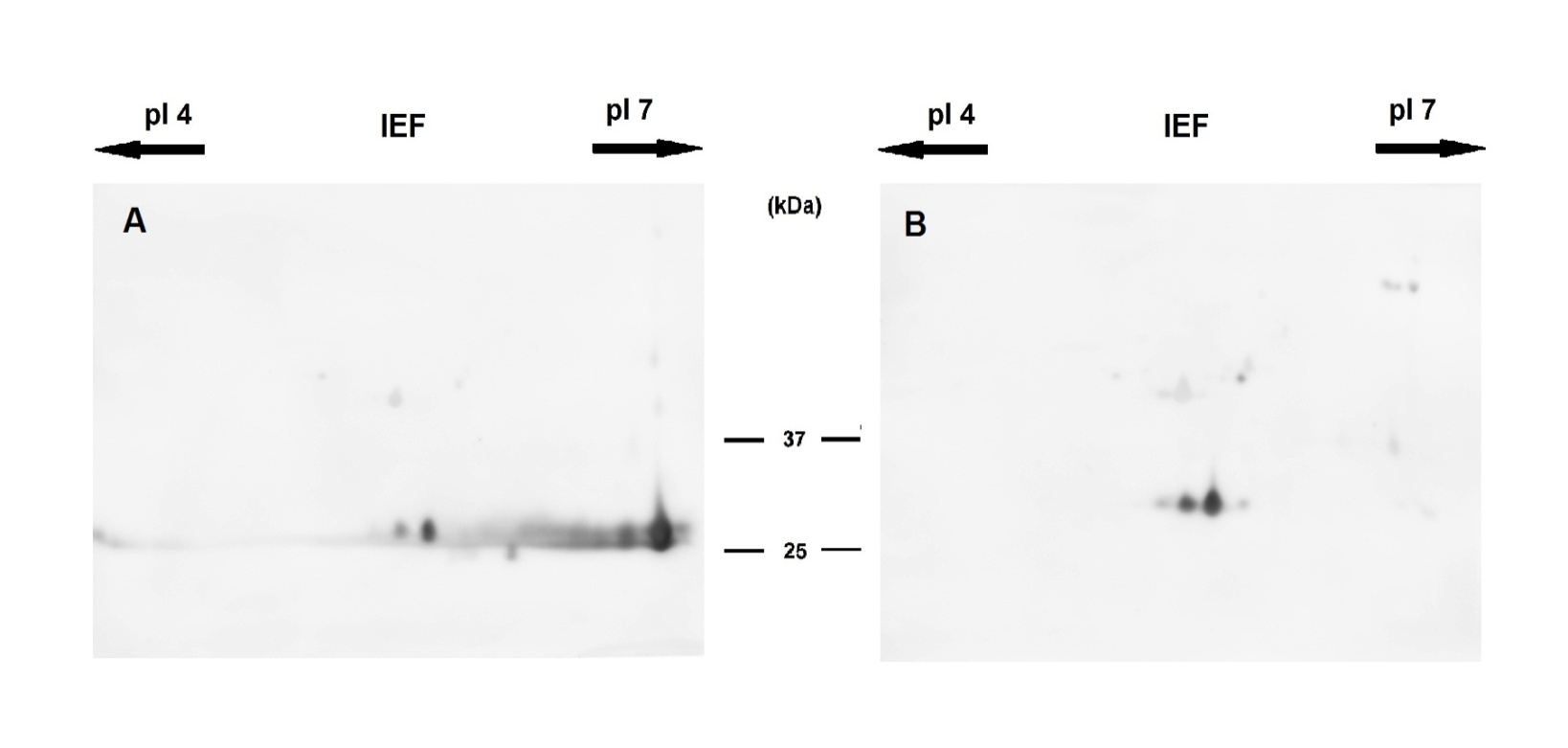


**Supplemental Figure 7**. 2-DE gel western blotting on erythrocyte samples (150 µg protein) of (A) ammocoete (N=4) and (B) post-metamorphic juveniles (N=4). Proteins were first separated by IEF in a pH gradient from 4 to 7 and further by SDS-PAGE (10%T). Membranes were probed using heterologous cytosolic carbonic anhydrase antibody (1:1000).


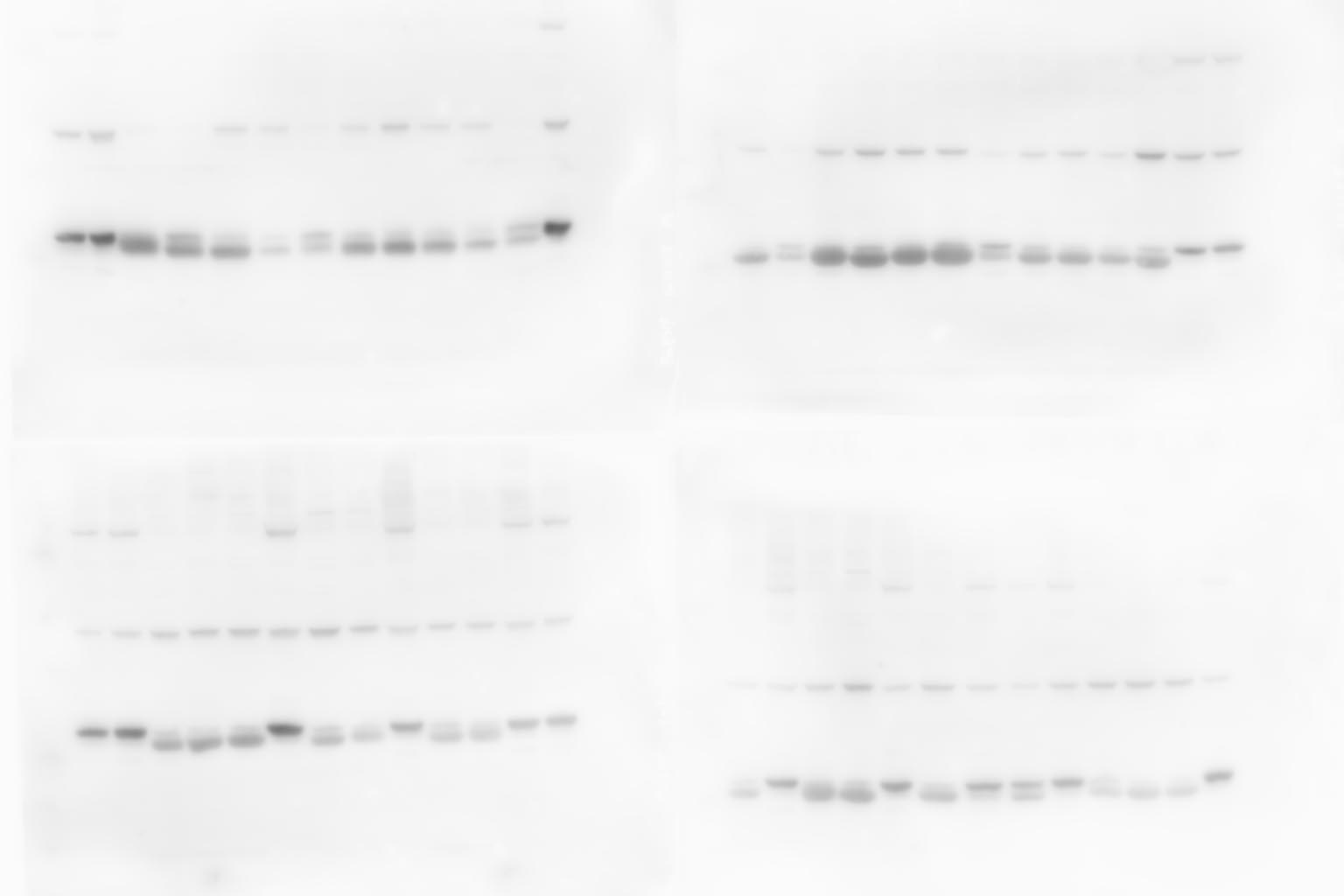


**Supplemental Figure 8**. Full western blotting membranes of gill samples from a metamorphic series probed using heterologous cytosolic carbonic anhydrase antibody (1:1000) and summarized in Fig 3.

**Supplemental References**

69. Rzhetsky, A. & Nei, M. A simple method for estimating and testing minumum-evolution trees. *Mol. Biol. Evol.* 9, 945–967 (1992).

70. Ferreira-Martins, D., Coimbra, J., Antunes, C. & Wilson, J. M. Effects of salinity on upstream-migrating, spawning sea lamprey, *Petromyzon marinus*. *Conserv. Physiol.* 4, cov064 (2016).

71. Qiu, Y. Lamprey Hemoglobin. Structural basis of the Bohr effect. *J. Biol. Chem.* 275, 13517–13528 (2000).

72. Neuhoff, V., Arold, N., Taube, D. & Ehrhardt, W. Improved staining of proteins in polyacrylamide gels including isoelectric focusing gels with clear background at nanogram sensitivity using Coomassie Brilliant Blue G-250 and R-250. *Electrophoresis* 9, 255–262 (1988).

73. Pandey, A. & Mann, M. Proteomics to study genes and genomes. *Nature* 405, 837–846 (2000).

74. Biasini, M. et al. SWISS-MODEL: modelling protein tertiary and quaternary structure using evolutionary information. *Nucleic Acids Res.* 42, W252–W258 (2014).

75. Arnold, K., Bordoli, L., Kopp, J. & Schwede, T. The SWISS-MODEL workspace: a web-based environment for protein structure homology modelling. *Bioinformatics* 22, 195–201 (2006).

76. Bordoli, L. et al. Protein structure homology modeling using SWISS-MODEL workspace. *Nat. Protoc.* 4, 1–13 (2009).

77. Dolinsky, T. J., Nielsen, J. E., McCammon, J. A. & Baker, N. A. PDB2PQR: an automated pipeline for the setup of Poisson-Boltzmann electrostatics calculations. *Nucleic Acids Res.* 32, W665–W667 (2004).

78. Pancer, Z. et al. Somatic diversification of variable lymphocyte receptors in the agnathan sea lamprey. *Nature* 430, 174–180 (2004).

79. Stock, D. & Whitt, G. Evidence from 18S ribosomal RNA sequences that lampreys and hagfishes form a natural group. *Science.* 257, 787–789 (1992).

80. Consoli, L. & Damerval, C. Quantification of individual zein isoforms resolved by two-dimensional electrophoresis: Genetic variability in 45 maize inbred lines. Electrophoresis 22, 2983–2989 (2001).
